# Infection phenotypes of a coevolving parasite are highly diverse, structured, and specific

**DOI:** 10.1111/evo.14323

**Published:** 2021-08-30

**Authors:** Maridel Fredericksen, Camille Ameline, Michelle Krebs, Benjamin Hüssy, Peter D. Fields, Jason P. Andras, Dieter Ebert

**Affiliations:** ^1^ Department of Environmental Sciences, Zoology University of Basel CH‐4051 Switzerland; ^2^ Department of Biological Sciences, Clapp Laboratory Mount Holyoke College South Hadley Massachusetts

**Keywords:** Antagonistic coevolution, cladocera, firmicutes, host–parasite interactions, pathogen, red queen

## Abstract

Understanding how diversity is maintained in natural populations is a major goal of evolutionary biology. In coevolving hosts and parasites, negative frequency‐dependent selection is one mechanism predicted to maintain genetic variation. While much is known about host diversity, parasite diversity remains understudied in coevolutionary research. Here, we survey natural diversity in a bacterial parasite by characterizing infection phenotypes for over 50 isolates in relation to 12 genotypes of their host, *Daphnia magna*. We find striking phenotypic variation among parasite isolates, and we discover the parasite can infect its host through at least five different attachment sites. Variation in attachment success at each site is explained to varying degrees by host and parasite genotypes. A spatial correlation analysis showed that infectivity of different isolates does not correlate with geographic distance, meaning isolates from widespread populations are equally able to infect the host. Overall, our results reveal that infection phenotypes of this parasite are highly diverse. Our results are consistent with the prediction that under Red Queen coevolutionary dynamics both the host and the parasite should show high genetic diversity for traits of functional importance in their interactions.

## Introduction

A major challenge in evolutionary biology is to characterize selective forces maintaining genetic diversity. According to the Red Queen hypothesis of coevolution, time‐lagged negative frequency‐dependent selection (NFDS) imposed by coevolving parasites is a powerful mechanism to promote and maintain genetic diversity in host populations (Clarke 1976; Hamilton 1980; Woolhouse et al. 2002; Nuismer 2017). Indeed, selection from parasites has been demonstrated to sustain host genetic diversity in a variety of systems (Brockhurst et al. 2004; Koskella and Lively 2009; Bérénos et al. 2011; King et al. 2011; Morran et al. 2011).

In a coevolving host–parasite system under Red Queen dynamics, NFDS is expected to operate on both antagonists, meaning not only host diversity, but also parasite diversity should be maintained. Many putatively coevolving host–parasite systems have demonstrated genetic diversity in the host (for example: Chaboudez and Burdon 1995; Koskella and Lively 2009; Lenz et al. 2013; Vergara et al. 2014; Bonneaud et al. 2018; Alves et al. 2019), whereas much less is known about the diversity of the coevolving parasites, especially regarding traits under selection. A few studies support the idea of high diversity in parasites: In the trypanosome–bumblebee system (*Crithidia bombi*–*Bombus terrestris*), high genetic diversity has been demonstrated in the parasite (Schmid‐Hempel et al. 2019). *Arabidopsis thaliana* and its pathogen *Pseudomonas* both show high genetic diversity, apparently maintained over long time periods (Karasov et al. 2018). High genetic diversity in both antagonists has also been observed using experimental host–parasite coevolution in the laboratory (Papkou et al. 2019). To test whether NFDS drives antagonistic coevolution in a given system, one must characterize genetic diversity at coevolving genes in both the host and the parasite (Lythgoe and Read 1998). For this, we need to understand how host and parasite diversity is functionally linked, that is, identify the interacting phenotypes, as this is the level on which selection acts.

In the water flea, *Daphnia magna*, and its bacterial parasite, *Pasteuria ramosa*, multiple lines of evidence suggest that this system undergoes Red Queen coevolutionary dynamics (Decaestecker et al. 2007; Ebert 2008; King et al. 2013; Auld et al. 2016). A genetic model of resistance has been developed through QTL mapping, GWAS analyses, and genetic crosses (Luijckx et al. 2013; Bento et al. 2020, 2017; Bourgeois et al. 2017; Ameline et al. 2021). This model includes several host loci with strong effects and epistatic interactions and was built based on the interaction of defined host genotypes with a small panel of *P. ramosa* isolates. To infect, the parasite must attach to the host cuticle at attachment sites in the foregut (esophagus) or the hindgut (Duneau et al. 2011; Ebert et al. 2016; Bento et al. 2020). Susceptibility/resistance can be scored by assessing whether parasite transmission stages (spores, which are fluorescently labeled for visualization) attach to the host or not. So far, our knowledge of diversity in *Daphnia–Pasteuria* interactions is highly skewed toward the host, because host clones are easily produced and propagated in the laboratory. If we are to truly understand the coevolution of this system, we must expand our surveys to include more parasite genotypes.

Previous studies hinted that we have only characterized a fraction of the variation in the parasite's infectivity. A microsatellite study of isolates from 25 populations across Europe and Western Asia revealed high levels of parasite genetic diversity within and between populations (Andras et al. 2018). Subsequently, variation in attachment to the foregut of one *D. magna* clone was pinpointed to two *P. ramosa* haplotypes, differing at seven amino acids in the *Pasteuria* collagen‐like (PCL) gene PCL7 (Andras et al. 2020). The *P. ramosa* genome contains over 50 such PCL genes, which are thought to have diversified in response to antagonistic coevolution (Mouton et al. 2009; McElroy et al. 2011). The expansion and diversification of this gene family suggests that phenotypic diversity of *P. ramosa* attachment could be vast indeed.

Our aim in the current study was to characterize the diversity of infection phenotypes (infectotypes) of over 50 *P. ramosa* isolates from several populations in relation to 12 *D. magna* host clones. We find that spore attachment can occur at several previously unknown sites. Furthermore, our results show that *P. ramosa* attachment phenotypes are strikingly diverse and that distinct clusters of similar phenotypes exist. Finally, similarity in attachment phenotypes correlates with genetic similarity and, to a weaker extent, geographic distance. We discuss the implications of these findings in the context of host–parasite coevolution.

## Methods

### HOST AND PARASITE GENOTYPES

A total of 61 isolates of *P. ramosa* were collected from localities throughout the Holarctic (Andras et al. 2018) as well as several isolates from a single population in Switzerland (Supporting information Table S1). Isolates were obtained either directly from infected field‐collected *D. magna* or by exposing host genotypes (clones) in the lab to field‐collected pond sediments, which harbor parasite resting spores (Ebert et al. 1996; Andras and Ebert 2013). We propagated each isolate for at least two infection cycles (serial passage) to reduce genotype diversity within isolates, because *P. ramosa* cannot be cultured in vitro (Luijckx et al. 2011). Isolates were propagated by homogenizing infected host tissue and exposing healthy hosts to this spore suspension (Ben‐Ami et al. 2010; Andras and Ebert 2013). Whole‐genome sequencing of *P. ramosa* isolated in the same way suggested that after two serial passages, the isolates consist mostly of one single genotype (Andras et al. 2018). Infected hosts were frozen 6−8 weeks after infection, shortly before parasite‐induced death. The *D. magna* clones that we used for spore propagation, attachment tests, and infection tests were also collected from various locations across their Holarctic range (Supporting information Table S1) and are maintained in the Ebert lab as part of the *D. magna* Diversity Panel (Seefeldt and Ebert 2019; Bento et al. 2020) under standard laboratory conditions (20°C, 16:8 day:night cycle, *Scenedesmus* sp. as food) (Ebert et al. 1998).

*Daphnia magna* clones can be classified by resistance phenotype (resistotype), scored as their binary attachment phenotype to a given set of *P. ramosa* isolates. In previous work, five isolates were mainly used to define resistotypes in this system (Metzger et al. 2016a; Bento et al. 2017, Bento et al. 2020; Ameline et al. 2021). For the current study, we defined infection phenotypes (infectotypes) for each *P. ramosa* isolate, that is, attachment of a parasite isolate to a defined set of host genotypes. Based on resistotype information from previous studies (Bento et al. 2020; Ameline et al. 2021), we chose 12 unique *D. magna* clonal lines to represent the infectotype panel. Among these 12 clones, one clone (HU‐HO‐2) was the subject of previous infectivity studies (Sison‐Mangus et al. 2018; Andras et al. 2020), three clones (the “CH‐H” clones) represent the most common resistotypes in the Aegelsee population in Switzerland (Ameline et al. 2021), which has been intensively studied over the past decade (Andras and Ebert 2013; Ameline et al. 2021). The other eight clones represent diverse resistotypes and populations of origin across Western Eurasia, East Asia, and North America (Supporting information Table S1).

### INFECTIVITY PHENOTYPING

**Figure 1:**
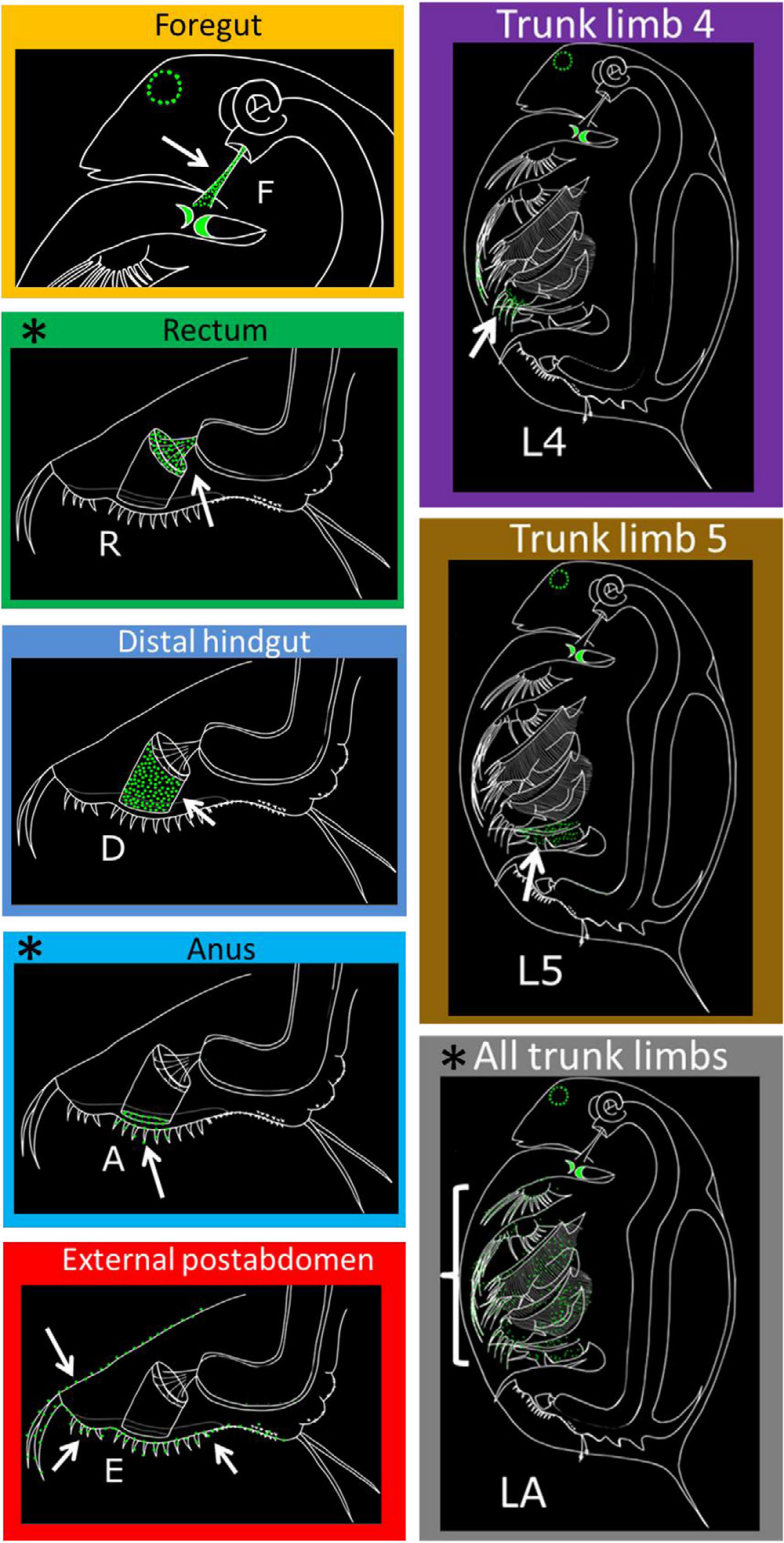
Schematic depiction of attachment of *Pasteuria ramosa* isolates to different sites on the cuticle of *Daphnia magna* hosts. Illustrations of infected *D. magna* as would be observed under fluorescence microscope. Green spots indicate fluorescently labeled *P. ramosa* spores and autofluorescence of host mandibles and compound eye. White arrows indicate the location of spore attachment to the host cuticle. Sites are referred to by the abbreviated names shown (F–LA). Asterisks indicate attachment sites that were rare or confounded with other sites (see text). These * marked sites were removed from the final dataset and the subsequent analyses.

Infection of *Daphnia* by *P. ramosa* follows several steps, of which attachment to the host's cuticle is most important with regard to infectivity (Ebert et al. 2016). We scored attachment phenotypes for parasite isolate–host clone combinations using the attachment test (Duneau et al. 2011). We extended previous work, by systematically screening each host for attachment of fluorescently labeled spores on the entire body. The final set of microscopically distinguishable attachment sites were scored as follows: The foregut (F), or esophagus, was scored as described previously, whereas the hindgut site was subdivided into three sites: rectum (R), distal hindgut (D), and anus (A) (in order from cranial to caudal). Additionally, we scored attachment to the external postabdomen (E) as well as the fourth trunk limb (L4), fifth trunk limb (L5), and all trunk limbs (LA), as shown in Figure 1 (see Supporting information for additional descriptions). For each attachment site, every individual *D. magna* received a binary (yes/no) score. Previous attachment tests showed foregut attachment scores were highly consistent among replicates of the same host‐parasite combination, whereas other sites were less consistent. Therefore, we assessed at least three *D. magna* individuals for each unique host–parasite combination, and for ambiguous phenotypes we increased the number of replicates up to 42. All attachment tests were scored by the same researcher.

We assessed repeatability of attachment scores in two ways. First, we examined the attachment phenotypes of our five *P. ramosa* “laboratory isolates” C1, C19, P15, P20, and P21, which have previously been tested for foregut (F) and hindgut (now distinguished as R, D, or A) attachment. We compared our current attachment scores with those produced by other researchers for previous studies (Bento et al. 2020, 2017), though only in relation to the foregut and hindgut sites. Second, for the data generated in the current study, we assessed repeatability using the residual variance component from a variance component analysis (see *Site‐specific attachment phenotype analysis* below).

Infectivity phenotyping revealed that some *P. ramosa* isolates showed identical attachment patterns for the specified 12‐host panel (Supporting information Figs. S1 and S2). Heatmaps were made to visually compare the attachment patterns among isolates (heatmap function in R version 4.0.3) (R Core Team 2020). Specifications included the Euclidean distance calculation and hierarchical clustering with the complete agglomeration method. Some of the duplicate isolates originated from the same population, in which case one isolate was arbitrarily selected to remain in the dataset, and duplicates were excluded from further analyses (Supporting information Fig. S2). Ten of the initial 61 isolates were removed during this deduplication step.

### INFECTION TEST OF NEW ATTACHMENT SITES

Attachment to the foregut and hindgut leads to infection (Duneau et al. 2011; Bento et al. 2020). Here, we tested whether infection also results from attachment to the newly described sites. We selected host–parasite combinations showing strong attachment (>50% of individuals tested showed a positive result) to our focal site and weak or no attachment to other sites. This requirement excluded sites R, A, and LA from testing because they could not be studied in isolation from other sites. For each of the other novel sites, we were able to select from six (site L4) to 12 (site E) host–parasite combinations. We also included two host–parasite combinations for each of the two previously studied attachment sites (foregut and distal hindgut) (Duneau et al. 2011; Bento et al. 2020). We included the host clone in which we initially propagated the given parasite isolate (as a positive control), and we included a host clone without attachment to the same parasite isolate (resistant, i.e. negative control). Unexposed hosts were included as a further control.

For each host–parasite combination, we had three jars (replicates) with cultures of the test animals (five females per 100‐mL jar), one jar of animals for the unexposed treatment, and one jar of animals from the passage host (positive control, Supporting information Fig. S3). For four host–parasite combinations, we had three jars containing resistant animals (negative control). Our infection procedures followed Luijckx et al. (2011, 2014). Host animals were 3−5 days old at time of exposure. Animals in each parasite‐exposure treatment received 75,000 spores on two consecutive days, for a total of 150,000 spores per animal. Animals received 3 million cells per individual of *Scenedesmus sp*. algae daily until day seven, 4 million until day 14, and 5 million afterwards. Animals were monitored daily for mortality, and those showing signs of infection were tested immediately for the presence of *P. ramosa*. All remaining animals were dissected from day 21 to 23.

### ATTACHMENT PHENOTYPE ANALYSIS

We used multiple methods to characterize the structure of our phenotypic data. We used the attachment test data to generate an overall attachment phenotype (which we define in relation to 12 host clones at the five sites shown to lead to infection) of each isolate. We conducted a principal component analysis (PCA) to determine which traits (combinations of host and attachment site) contributed most to the variation in this overall attachment phenotype (prcomp function in R, with center = TRUE and scale = FALSE).

We conducted a variance component analysis for each site‐specific attachment phenotype and a consensus attachment phenotype to assess the relative contribution of host clone versus parasite isolate to the variation in each attachment phenotype, fitting binary random‐effects models of the form attachment ∼ host + parasite + host*parasite + error with a logit link, using the function observGlmer in the R package *fullfact*, version 1.3 (Houde and Pitcher 2016).

We assessed the potential for nonrandom associations between attachment sites in our dataset. We tallied host–parasite combinations (out of 612 total) that showed binary positive attachment scores (>50% positive scores among replicates) at zero, one, two, and three or more sites. We then compared these observed counts to the counts expected based on the frequency of each attachment site in our dataset using a chi square goodness‐of‐fit test. Additionally, we used 2 × 2 contingency tables evaluated with Fisher's exact test to determine whether parasite attachment to pairs of sites in the same host clone was observed more or less often than expected by chance. The *P*‐values were evaluated at alpha = 0.005 (after Bonferroni correction for 10 tests).

### DISTANCE COMPARISONS

We compared the genotypic, phenotypic, and geographic pairwise distances for a subset of 22 *P. ramosa* isolates for which we had genomic data (Andras et al. 2020). Genotypic distances were calculated as kinship coefficients from genome‐wide SNP data as described previously (Andras et al. 2020). Phenotypic distances were calculated as Euclidean distances using our quantitative attachment matrix (Supporting information Fig. S1, which considers proportion of tested host individuals that showed spore attachment), with only the five attachment sites that lead to infection (i.e. sites F, D, E, L4, L5). We additionally tested a binary consensus phenotype representing each parasite's overall infectivity to each host clone, regardless of attachment site. We compared each pairwise distance (geography:genotype, genotype:phenotype, geography:phenotype). For the genotype:phenotype comparison, we used a Mantel test, evaluating whether Mantel's r (Mantel 1967) was different from zero, using 100,000 permutations (R package *ecodist*, v2.0.7 [Goslee and Urban 2007]). Use of the Mantel test in spatial analyses has been critiqued (Legendre et al. 2015) in favor of the more accurate distance‐based Moran's eigenvector (dbMEM) analysis. For this reason, we used the dbMEM method for the comparisons that included geographic distance (geography:genotype and geography:phenotype). We performed a dbMEM analysis (Legendre et al. 2015) by redundancy analysis (R packages *adespatial* [Dray et al. 2020] and *vegan* [Oksanen et al. 2020] as described previously [Andras et al. 2018]).

## Results

### NEWLY DESCRIBED ATTACHMENT SITES

We surveyed 61 *P. ramosa* isolates from 19 populations in nine countries by testing the isolates against a panel of 12 host clones (Supporting information Fig. S1, Tables S1, S2). Parasite spores were found to attach to the host cuticle at eight sites (Fig. 1, see supplemental material for images), of which only the foregut and hindgut sites had been described previously (Duneau et al. 2011; Bento et al. 2020). Based on the phenotypes, we subdivide hindgut attachment into three distinct sites (from cranial to caudal: rectum (R), distal hindgut (D), and anus (A)), and we describe four new attachment sites: external postabdomen and three distinct limb attachment phenotypes: trunk limb 4 (L4), trunk limb 5 (L5), and all trunk limbs (LA).

Repeatability of the attachment tests was generally high. A comparison of new data with previously obtained data generated by a different investigator suggested a repeatability of approximately 95% (n = 78 attachment phenotypes tested) for foregut and hindgut attachment (Supporting information Fig. S4A). Likewise, variance component analyses with the new data revealed that, depending on the attachment site, only 3 to 22% of the variation in attachment success was unexplained (residual variance) (Supporting information Fig. S4B). Sites F, E, L4, and L5 had below 5 %, while site R stood out with 22 % (Supporting information Fig. S4B).

### INFECTION OCCURS THROUGH MULTIPLE SITES ON THE HOST CUTICLE

**Figure 2:**
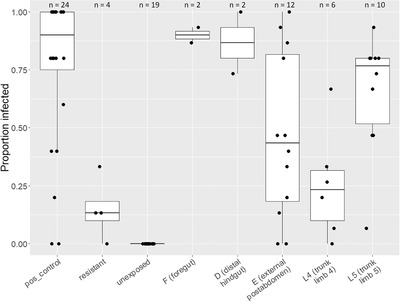
Boxplot showing proportion of infected hosts resulting from *Pasteuria ramosa* spore attachment to various sites on *Daphnia magna* cuticle. Box edges denote first and third quartiles, and whiskers extend to 1.5 × interquartile range. Points represent host clone–parasite isolate combinations, with 15 host individuals (for the resistant group and test groups F–L5) or five host individuals (for positive control and unexposed groups) tested per combination. Number of combinations tested (n) are given above each treatment group. For the test groups (F–L5), we specifically chose host–parasite combinations that show strong attachment at only the site being tested. For the positive control group, each tested *P. ramosa* isolate was paired with the host in which it was previously propagated. For the resistant group, we tested host–parasite combinations which showed no attachment at all sites.

We tested the infection potential of each attachment site using host–parasite combinations that showed strong attachment at only one site. Our infection trials revealed that attachment to sites E, L4, and L5 can lead to infection (Fig. 2), with L5 leading most reliably to infection (10 of 10 replicates showing infections). Furthermore, three replicates showed exclusive attachment to site L5 (Supporting information Fig. S5A), indicating strong support that this site leads to host infection. Site L4 showed lower infection overall compared to L5 (Fig. 2). Even so, one out of six replicates showed exclusive attachment to L4, and 33% of these hosts became infected (Supporting information Fig. S5A). Site E showed high variation in infection success (Fig. 2). This site also proved difficult to isolate, with only one out of 12 hosts showing exclusive attachment to the focal site (Supporting information Fig. S5A). This replicate did show 100% infection though, indicating that infection can occur through the external postabdomen. All other replicates showed the highest attachment to the focal site (red cells in “E” column, Supporting information Fig. S5A).

Attachment to sites F and D was reliably high (>90%, Supporting information Fig. S5A) and showed high infection rates (>70%, Fig. 2), which is consistent with previous studies (Duneau et al. 2011; Luijckx et al. 2011; Bento et al. 2020). Host clones without detectable attachment (presumably resistant) showed a median infection rate of 13% (Supporting information Fig. S5B), suggesting additional, but less efficient, attachment sites may await discovery. Attachment and infection results from the positive control group followed our expectation (Supporting information Fig. S5C).

Our current dataset did not allow us to test infectivity of attachment sites R (rectum), A (anus), and LA (all trunk limbs), as we were unable to separate these sites from other sites (for example, site R covaries with site D, Supporting information Fig. S6). Similarly, sites A and LA are ambiguously defined in relation to other sites. One isolate (P27) attached only to site LA, and attachment was below threshold for all hosts at all sites. This suggests the 50% attachment threshold may be too high to define susceptibility in some cases (Supporting information Fig. S7), or additional attachment sites may await discovery.

### DIVERSE INFECTOTYPES

**Figure 3:**
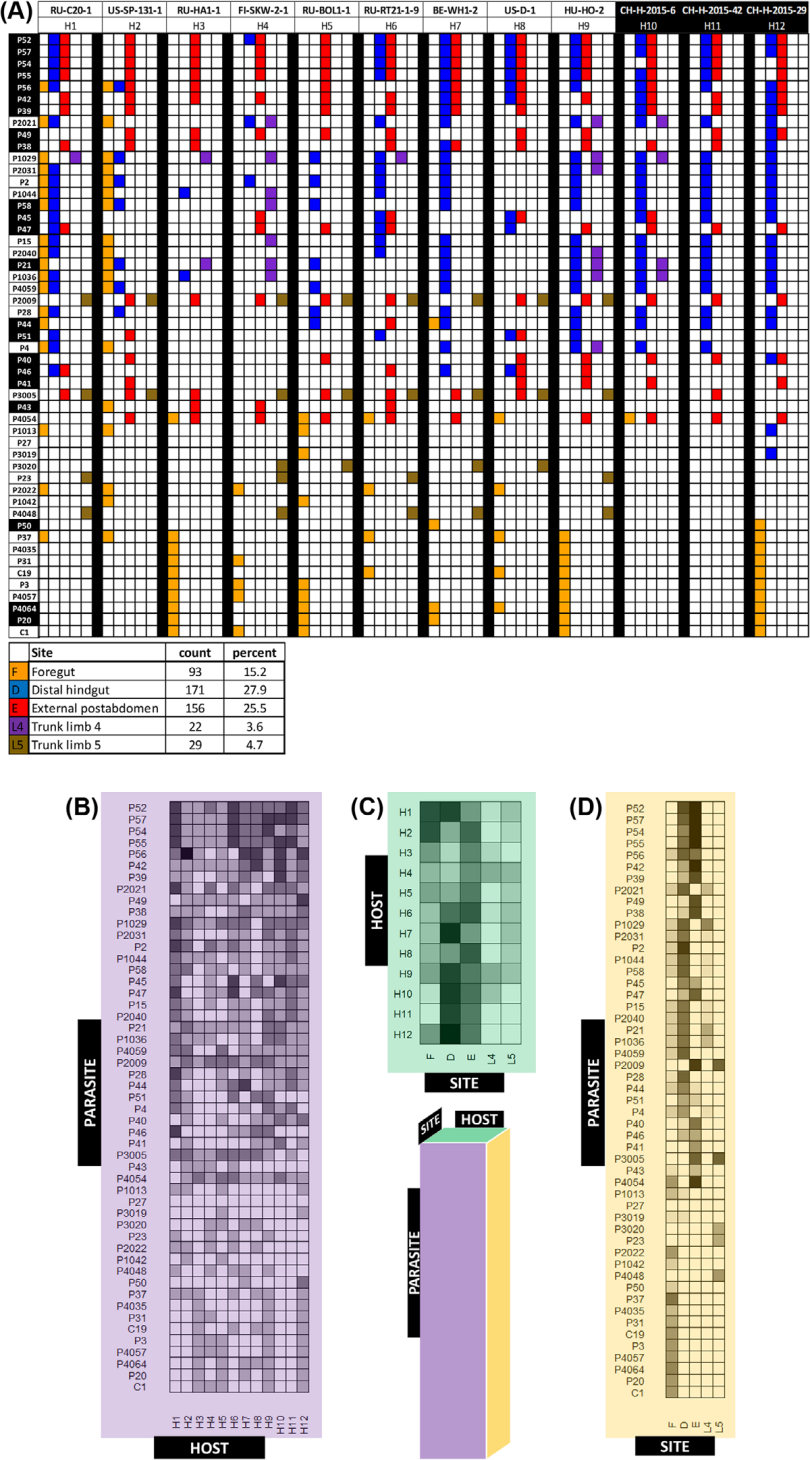
Parasite–host predicted infectivity matrices**. (A)** Binary attachment results (infective vs noninfective) for 51 *Pasteuria ramosa* isolates, 12 host clones, and five attachment sites. Parasite duplicates and attachment sites that did not lead to infection (see Fig. 2) were removed. Each colored square represents an attachment test for which >50% of tested host individuals showed attachment. Parasite isolates are sorted from top to bottom according to their order along principal component 1 (see Fig. 4). Host and parasite labels with white text on black background indicate samples from the Aegelsee population in Switzerland. Matrix was generated using the R package *plot.matrix*, version 1.4 (Klinke 2020). The summary table below the matrix shows counts and percentages of positive attachment at each site. **(B–D)** Consensus matrices representing two‐dimensional versions of the full three‐dimensional (parasite × host × site) matrix in (A), with mean attachment scores calculated across each axis. Each cell is shaded according to the proportion of positive attachment tests for each given combination. **(B)** Parasite–host consensus matrix (mean across attachment sites). Each cell represents one pairing of *Daphnia magna* clone (columns) and *Pasteuria ramosa* isolate (rows). **(C)** Host–site consensus matrix (mean across parasite isolates). Rows represent host clones and columns represent attachment sites. **(D)** Parasite–site consensus matrix (mean across hosts). Rows represent parasite isolates and columns represent attachment sites.

We analyzed the diversity of our isolates by first considering attachment at all infective sites together as one composite phenotype (i.e. composed of several traits). For this, we defined an infectotype for each *Pasteuria* isolate from the combination of the five attachment sites that led to infection (Fig. 3A). We set a threshold of 50% attachment to distinguish isolates predicted to cause infection (“infective isolates”) from isolates predicted to not cause infection (“noninfective isolates”), (following Bento et al. 2020). We also summarized the infectivity matrix (Fig. 3A) in the form of three consensus matrices (Fig. 3B–D). These result from averaging the three‐dimensional (parasite × host × site) matrix along each of its axes and representing the data as quantitative (Fig. 3B–D) or binary (Supporting information Fig. S8). Biologically, the consensus matrices represent the parasite infectivity to each host (Fig. 3B, Supporting information Fig. S8A), host susceptibility at each site (Fig. 3C, Supporting information Fig. S8B), and parasite infectivity at each site (Fig. 3D, Supporting information Fig. S8C).

Among the 51 isolates in our dataset, we found 48 isolates with unique infectotypes including 22 unique infectotypes from a single population in Switzerland (Aegelsee, marked in black in Fig. 3A). Moreover, a total of 41 isolates had a unique consensus infectotype (Fig. 3B), which disregards the attachment site and considers only whether the host—parasite combination is compatible (i.e. leads to infection). These findings indicate high phenotypic diversity in *P. ramosa* across Eurasia and even within a single population.

Parasite isolates varied in their host ranges and attachment site specificities (Fig 3B): five isolates attached to all 12 host clones, whereas one isolate did not attach to any host clones. In terms of attachment site specificity (Fig 3D), 11 isolates, including our long‐term laboratory work horses, isolates C1, C19, and P20, attached exclusively to site F, three isolates (P23, P3020, P4048) attached exclusively to site L5, and one isolate (P41) attached exclusively to site E. Other isolates attached to multiple sites, often within a single host. None of the isolates attached to all five sites; the maximum was three sites. Strikingly, all isolates except one (P56) attached to only one or two sites within the same host clone. Additionally, the total number of sites an isolate can attach to across hosts is positively correlated with the number of susceptible hosts this parasite can infect (Spearman's rho = 0.61, Supporting information Fig. S9), suggesting that isolates which can attach to more sites also have a wider host range, a form of generalist–specialist continuum.

All host clones were susceptible, but they varied in the number of parasite isolates to which they were susceptible, ranging from 24 to 41 out of a total of 51 isolates (Fig. 3B, Supporting information Fig. S8A). Most host clones were susceptible to spore attachment at most sites (Fig. 3C, Supporting information Fig. S8B), still some seem resistant to certain types of attachment, but our sample size might be too low to find rare attachment.

**Figure 4:**
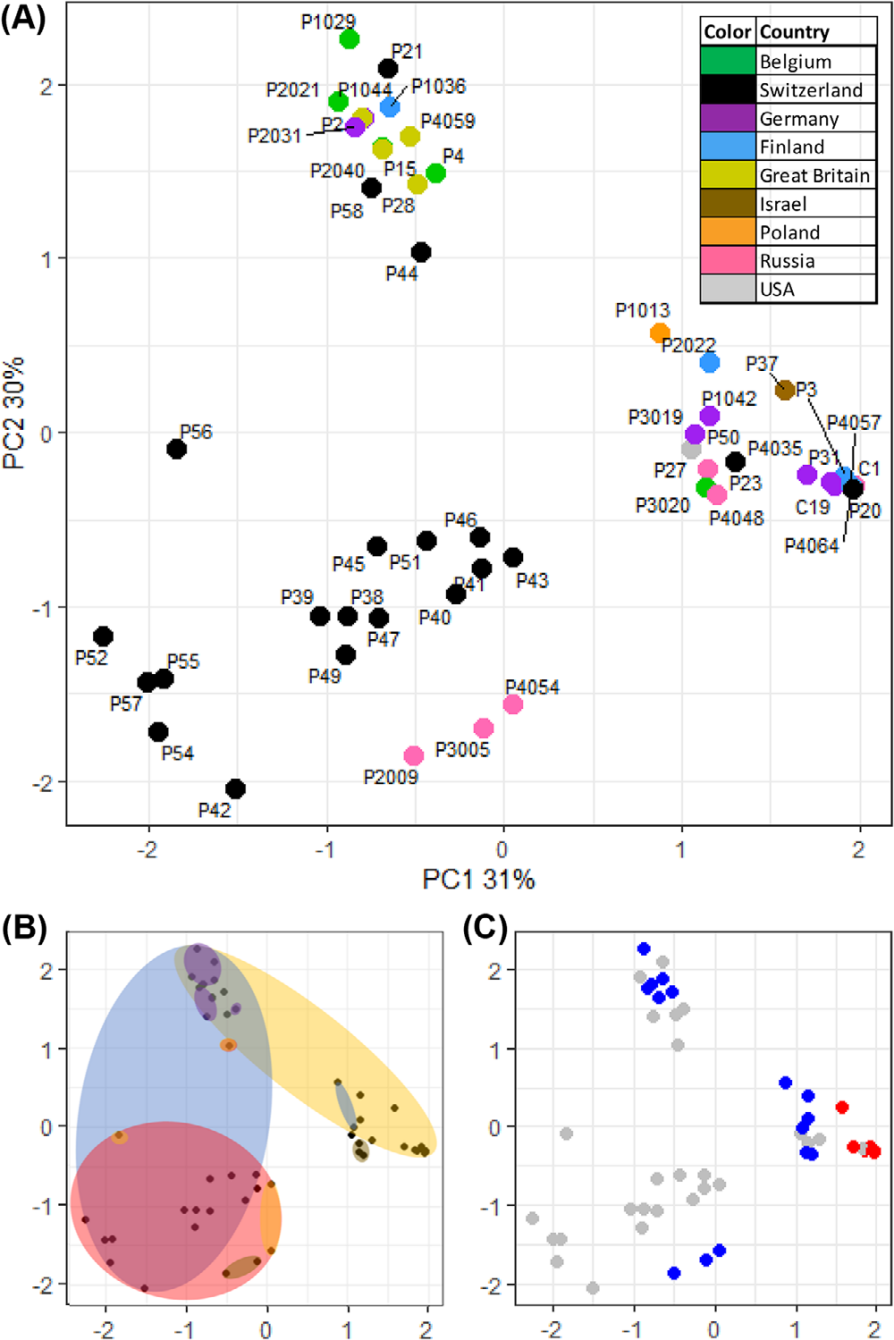
Parasite isolates cluster by infectotype. **(A)** Principle component analysis (PCA) plot of *P. ramosa* infectotypes using quantitative attachment data. Each point represents one of 51 *P. ramosa* isolates. Proximity along principle component axes 1 and 2 (PC1, PC2) indicates similarity in attachment to five attachment sites on 12 host clones. Colors indicate the country of origin. All points in black indicate the same population in Switzerland. Note that PC1 and PC2 together explain 61% of variation in the overall data, whereas the following PCs explain considerably less variation (PC3: 8%, PC4: 4%, PC5: 4%). Plots (B) and (C) show the data as in (A), but with different aspects highlighted in each. **(B)** Colored ellipsoids represent the area that encompasses the most points, representing isolates that attach to the given site on the host cuticle (colors as in Fig. 1: yellow = foregut, blue = distal hindgut, red = external postabdomen, purple = trunk limb 4, brown = trunk limb 5). **(C)** Polymorphism at PCL7 gene: red points denote isolates with the infective PCL7 haplotype, blue points denote isolates with the non‐infective PCL7 haplotype (Andras et al. 2020), and gray points denote isolates for which we do not have genetic data.

A principal component analysis showed that the *Pasteuria* phenotypes clustered well across PC1 and PC2, which together explained 61% of the variation in attachment phenotype (Fig. 4, see Supporting information Table S3 for loadings), while PC3 explained only 8 % of the variation. The 51 *P. ramosa* isolates show some clustering by attachment specificity (colors in Fig. 4B) but not by the population of origin. Each cluster contains isolates from diverse origins (Fig. 4A), and isolates from the Aegelsee population are found in each of the three major clusters. One tight cluster on the right (>1.5 on PC1, Fig. 4A) contains all isolates in our dataset that are known to possess the infective allele of the PCL7 gene, which was shown to confer attachment to the foregut of clone H9 (Fig. 4C) (Andras et al. 2020). Taken together, the attachment phenotypes of the 51 *P. ramosa* isolates show a high functional diversity, with similar phenotypes found in several widely separated places, and very different phenotypes occur in the same population.

### SITE‐SPECIFIC ATTACHMENTS SHOW NONRANDOM ASSOCIATIONS

The five infective sites showed varying patterns of attachment among the parasites and hosts sampled. Variance components differed strongly among sites (Table 1, Supporting information Fig. S10), with attachment to sites E and L5 mainly explained by parasite variation (>90%), while L4 and F attachment were primarily explained by host–parasite interactions (>90%). Surprisingly, across all sites, the host main effect was smallest, ranging from negligible effects to 14%. Residual variance was also consistently low (3−15%), which reflects our earlier result of high repeatability for the foregut and hindgut attachment phenotypes. We also calculated the variance components explaining binary consensus infectivity (attachment at any infective site, Supporting information Fig. S8A) and found strong effects of both parasite (32%) and host–parasite interactions (47%), while host effect and residuals were both low (6 and 15%, respectively).

**Table 1:** Attachment site‐specific attachment diversity

Percent variance					
	F	D	E	L4	L5
Effect	Foregut	Distal hindgut	External postabdomen	Trunk limb 4	Trunk limb 5
host	0	14	0.04	0	2.9
parasite	0	56	94.9	4.3	90.4
host × parasite	97.4	17.9	1	91.8	2.2
residual	2.6	12.1	4.1	3.9	4.4

Percent of attachment variance explained by host clone and parasite isolate main effects, host x parasite interactions, and residuals. Variance component analysis was performed in the R package *fullfact*, with host and parasite treated as random effects, and a binary response variable (attach/no attach) for each replicate tested.

The overall distribution in the number of attachment sites observed for each of the 612 host–parasite combinations differed from expectations based on the frequency of each attachment site in our matrix (Supporting information Fig. S11A, χ^2^ = 14.036, *P* = 0.00286). This result was mainly influenced by the rarity of attachment at three or more sites. Specific pairs of sites also showed deviations from randomness (Supporting information Fig. S11B). Four of the ten possible pairwise combinations showed deviations from expectations under a random distribution. Site combinations F&E, E&L4, D&L5 were underrepresented in our dataset, whereas site combination D&L4 was over represented.

### SPATIAL AND GENETIC VARIATION

**Figure 5:**
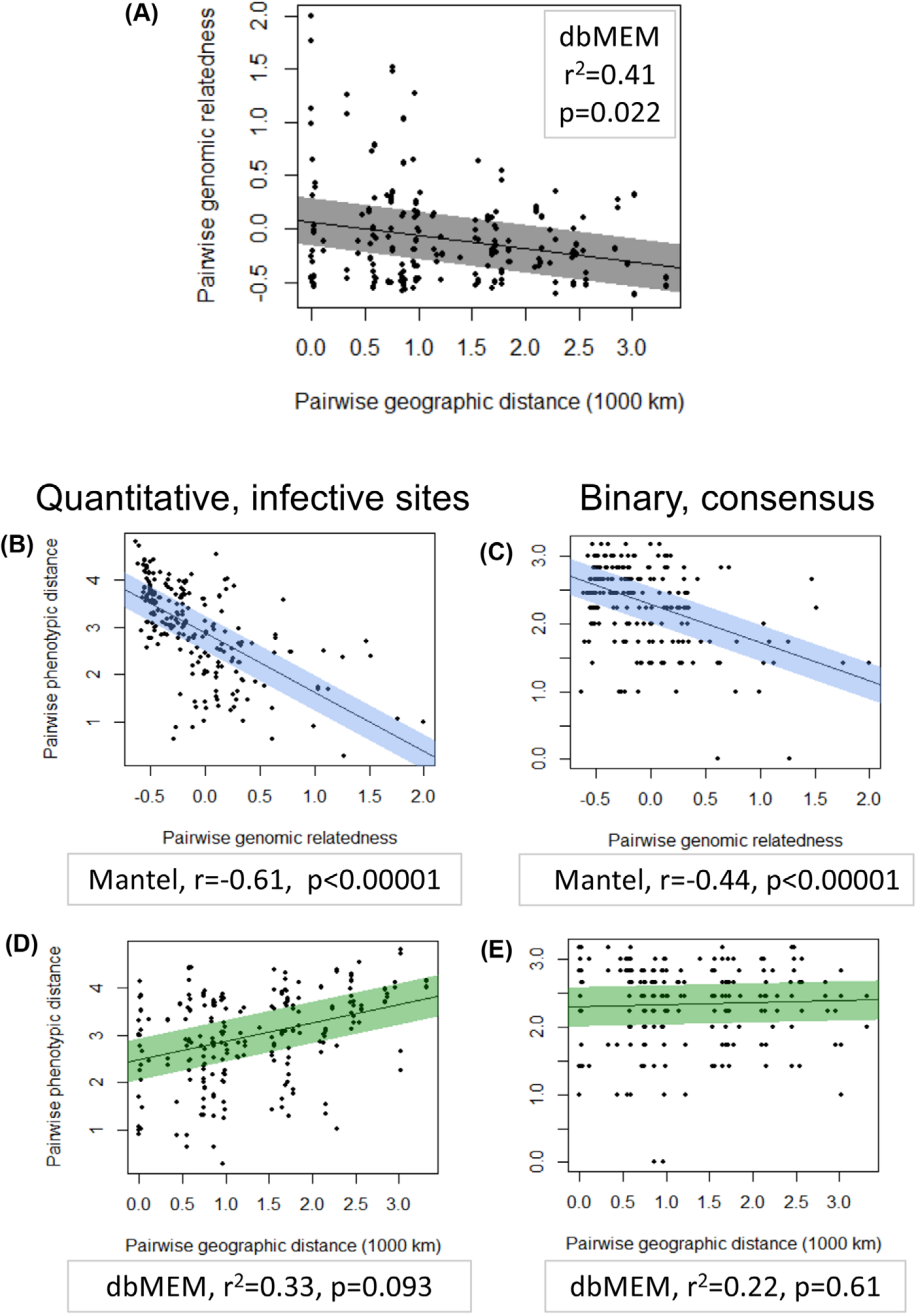
Distance matrices correlate. **(A)** Scatterplot showing relationship of pairwise whole genome relatedness (kinship coefficients) to pairwise geographic distances between sites of origin of populations for 22 *Pasteuria ramosa* isolates from our dataset (see Methods). Line of best fit was generated using a linear model. The shaded region spans 0.5 standard deviation of the residuals on either side of the line. **(B‐E)** Scatterplots showing relationship of pairwise Euclidean distances between attachment phenotypes to pairwise whole genome relatedness (B,C, blue shading) and to pairwise geographic distances between populations of origin (D,E, green). Phenotype is defined either as quantitative attachment to infective sites (B,D, left column) or as binary infectivity to each host regardless of attachment site (C,E, right column, data as in Supporting information Fig. S8). For each plot, the corresponding test, test statistics and *p*‐value is given.

To assess how well the overall attachment phenotype of an isolate is explained by its genotype as well as its geographic origin, we calculated correlations between the three matrices representing pairwise genomic relatedness, phenotypic distance (i.e. dissimilarity), and geographic distance. As expected from models of isolation by distance (IBD), pairwise geographic distance and pairwise genomic relatedness showed negative association (Fig. 5A, B: dbMEM *r* ^2^ = 0.41, *P* = 0.022), as was reported previously with a similar dataset (Andras et al. 2018). Genomic relatedness showed strong negative correlation with phenotypic distance calculated from quantitative attachment scores at infective sites (Fig. 5C, G: Mantel *r* = −0.61, *P* = 0.00001), meaning that isolates with similar attachment phenotypes also had more similar genomes. On the other hand, phenotypic distance and geographic distance showed a nonsignificant positive relationship (Fig. 5D, G: dbMEM *r* ^2^ = 0.33, *P* = 0.093). Together, these results indicate that geographic distance plays a weaker role compared to genetic distance in explaining variation in attachment phenotype.

We repeated the same analysis but with phenotypic distance calculated from the binary consensus matrix (Supporting information Fig. S8A). This matrix includes all available information for each host–parasite combination into one functional category; thus, it shows overall infectivity for each isolate, regardless of attachment site on the host. We included this additional analysis because overall infection outcome may be more under selection than the site of parasite entry into the host. From this analysis, we still found a strong correlation of phenotypic distance with genomic relatedness (Fig. 5E, G: Mantel *r* = −0.44, *P* = 0.00001), but we found a very weak, nonsignificant association of phenotypic distance with geographic distance (Fig. 5F, G: dbMEM *r* ^2^ = 0.22, *P* = 0.61). These results are consistent with the patterns suggested from our PCA analysis, namely that population of origin is a poor predictor of *P. ramosa* attachment success.

## Discussion

We characterized the functional diversity of a bacterial parasite in terms of infectivity to its host, a planktonic crustacean. We aimed to understand the often‐neglected parasite side of a natural system believed to coevolve by negative frequency‐dependent selection. Host genetic diversity is known to be high in such systems, whereas parasite diversity has rarely been addressed. Our expectation was, corresponding to the host's high genetic diversity for resistance, that the parasite would show high genetic diversity for infectivity. To this end, we experimentally assessed attachment phenotypes of 61 *P. ramosa* isolates in relation to eight possible sites on the cuticle of 12 host genotypes (clones of *D. magna*). These sites are possible entrance points of the parasite into the host's body cavity. Our data revealed high genetic diversity in attachment phenotypes across samples from three continents. We demonstrated that spore attachment to at least five of these sites can lead to infection. Groups of parasite isolates clustered in attachment specificity to combinations of hosts or attachment sites. These clusters had similar phenotypes but included isolates from distant geographic locations. On the other hand, isolates derived from a single population were found in diverse clusters. Our results are consistent with balancing selection on infection phenotypes in this bacterial parasite.

### DIVERSITY IS FUEL FOR, AND EVIDENCE OF, ONGOING COEVOLUTION

The functional diversity we observed among our 61 sampled isolates is striking. In total, 48 of our samples (79 %) showed unique infectotypes, and 22 of these unique isolates came from just a single population in Switzerland. The *D. magna* population in this pond is also known to have a high diversity of host resistotypes (Ameline et al. 2021). Thus, this population contains high variation in functionally coupled phenotypes of hosts and parasites, a hallmark of coevolution by negative frequency‐dependent selection (Lively 2010). This diversity is the basis of the Red Queen hypothesis, with genetic variation being both a driver and a consequence of coevolution.

High levels of parasite diversity have also been observed in other natural host–parasite systems predicted to be coevolving by negative frequency‐dependent selection, for example, in *Pseudomonas* infection of *Arabidopsis* (Karasov et al. 2018) and *Crithidia* parasites of bumblebees (Schmid‐Hempel and Funk 2004). As in the *D. magna–P. ramosa* system, the high diversity in *C. bombi* is thought to result from strong host–parasite interactions (Schmid‐Hempel et al. 2019).

### DIVERSITY IS STRUCTURED

While *P. ramosa* infectivity is diverse, it also shows clear structure. Our PCA analysis (Fig. 4) indicated that attachment site is an important distinguishing trait among isolates. Interestingly, the first two principal components explain the vast majority of phenotypic variation. The PCA clusters contained isolates from distant populations, and isolates from a single Swiss population (Aegelsee) were found in every major cluster. These observations suggest that diversity in the *P. ramosa* attachment phenotypes might be similarly structured across populations, which may indicate the presence of constraints limiting the dimensionality of phenotypic variation.

From these findings, we can derive several hypotheses about the genetic architecture of attachment specificity. First, the clustering, coupled with our finding that variation in the genome correlates to variation in attachment phenotype, suggests that groups of isolates share alleles underlying attachment at the respective sites. Indeed, isolates sharing a known variant of an infectivity allele at locus PCL7 (Andras et al. 2020) formed a tight cluster in our PCA plot (Fig. 4C). Second, the finding that clusters contained isolates originating from distant localities suggests these shared alleles are widely distributed geographically. Finally, the high variance explained by the first two principal components and the dense cluster of the PCL7 variants is consistent with the idea that infectivity alleles may occur at few genetic loci of major effect. Thus, we find strong evidence that genetic structure underlies the diversity in *P. ramosa* attachment success, that the dimensions of this genetic structure are limited, and that these limitations may have similar effects across populations. Future studies may investigate the isolates forming the PCA clusters in further analyses such as GWAS and proteomics.

### WEAK GEOGRAPHIC STRUCTURE IN INFECTION DIVERSITY

Our PCA revealed that similar infectotypes of *P. ramosa* isolates are found in distant localities and, consistent with this, our comparisons of genetic, phenotypic, and geographic pairwise distances suggest geographic distance is less important than genetic distance for explaining the diversity in *P. ramosa* infection phenotype. The strong association between genetic distance and phenotypic distance suggests that the PCA clusters are similar genetically, not only phenotypically. The weak association between geographic distance and phenotypic distance indicates *P. ramosa* attachment phenotype has a weak signal of IBD (Wright 1943). Two alternate explanations could give rise to such a weak IBD signal: infectivity could be caused by homologous alleles that spread between populations, or infectivity alleles could have evolved independently, at the same or different loci. So far, only one *P. ramosa* gene (PCL7) explaining an attachment polymorphism is known (Andras et al. 2020). The multi‐SNP polymorphism at this gene was shown to exist across distant populations, with the infecting PCL7 haplotype being of monophyletic origin; thus, it seems unlikely that similar attachment phenotypes evolve independently from each other in different populations. Rather, genes underlying attachment phenotypes may have spread widely.

Our genomic data showed a stronger pattern of IBD compared to the phenotypic data, suggesting that infectivity alleles have a higher effective migration rate compared to neutrally evolving alleles. Several natural host and parasite systems also show patterns of IBD (Barrès et al. 2008; Zieritz et al. 2010; Koop et al. 2014). The strength of IBD is inversely related to gene flow among populations (Sexton et al. 2014), but may be influenced by selection. If immigrant alleles have a local advantage, their effective migration rate would be higher than those of genes without a selective advantage. This is believed to be the case for alleles under balancing selection, which may enjoy an advantage of being rare upon arrival in a new population (Leducq et al. 2011). Thus, alleles underlying host resistance and parasite infectivity are expected to have higher effective migration rates than neutrally evolving alleles and thereby show a weaker pattern of IBD, that is, lower levels of geographic divergence (Ebert and Fields 2020). This is consistent with our results (Fig. 5). Alleles underlying *Pasteuria* infectotypes may have either spread more rapidly across populations compared to neutral alleles, or they have been conserved for longer as ancient polymorphisms, which has also been proposed for *D. magna* resistance alleles (Luijckx et al. 2014) (Y. Bourgeois, P.D. Fields, and D. Ebert, resubmitted to MBE).

While it seems likely that infectivity is caused by homologous alleles that spread through populations, either as recent polymorphisms or ancient polymorphisms, some aspects of attachment phenotype may have evolved independently. The weak link between geographic distance and phenotypic distance was especially striking when we collapsed variation in the specific site of attachment and considered only the overall (consensus) attachment to each host clone: in this case, the association between geography and phenotype entirely disappeared (Fig. 5F). Since selection acts on overall infectivity in the parasite, the consensus infectotype might be the more evolutionarily relevant version of the attachment phenotype. Thus, the finding that geographic distance correlates (weakly) with attachment phenotype but not with infection phenotype suggests that *P. ramosa* isolates from around the world have similar abilities to infect diverse hosts, although the route of infection may vary between populations. This may mean that infectivity to some host populations has convergently evolved: *Pasteuria* isolates from different populations may have solved the same problem (infecting a host) using different strategies (different routes of entry).

### DIVERSITY IS SPECIFIC

Site‐specific attachment phenotypes were explained to varying degrees by host effects, parasite effects, and host × parasite interaction effects. These distinct effects created distinct attachment patterns and suggested that attachment at each site may be mediated by different underlying genes. Genotype × genotype (G × G) interactions are at the core of coevolutionary theory (Thompson 1994; Nuismer 2017). Indeed, we observed strong host × parasite (G × G) interactions explaining attachment to three of the attachment sites: F, D, and L4. These G × G interaction are a hallmark of the *Daphnia–Pasteuria* system, and they have been highlighted in earlier studies for attachment to sites F and D as well as overall infection success (Carius et al. 2001; Duneau et al. 2011; Luijckx et al. 2011; Bento et al. 2020). However, not all sites showed host × parasite interaction effects. Attachment to the external postabdomen (site E) was explained almost entirely by parasite genotype and not by host genotype, raising the question, why have not all *P. ramosa* isolates evolved the ability to attach there? For one, multisite attachment may be costly. Parasite isolates in our sample attached to maximum three out of five sites, so perhaps expanding the number of sites to attach comes with increasingly higher costs for the parasite. Additionally, different attachment sites may vary in their suitability as entry routes to the host body. The high variability in infection success at site E suggests this site might be an unreliable point of entry for spores, which stands in contrast to sites F and D (Fig. 2). This difference may relate to the sclerotization of *D. magna*’s postabdomen, being a rather stiff structure with a role in cleaning the filter apparatus and possibly in predator defense. Sclerotization may reduce the ability of the parasite spores to penetrate the cuticle, reducing overall infection success. The cuticle at the foregut (F) and the hindgut (D) are not sclerotized. Consistent with this mechanical role of the cuticle in parasite defense, Izhar et al. (2020) found that penetration of the host cuticle takes substantially longer in older and larger hosts, which presumably have a thicker cuticle. If there is indeed a limited ability to attach to multiple sites, selection may favor parasites that attach to site F or D over site E, when hosts susceptible at sites F and D are common.

Besides our observation that parasite isolates attached to maximum three out of five sites, we also observed pairs with double‐positive attachment being either over‐ and underrepresented. Such nonrandom associations can arise through genetic interactions, such as physical linkage, pleiotropy (one locus influences multiple attachment phenotypes), and epistasis (multiple loci influence the attachment at one site), limiting the range of potential attachment combinations. The same was reported for host resistance. At least one host resistance locus is known with a pleiotropic effects on foregut and hindgut attachment (Bento et al. 2020) and different loci are known to interact epistatically (Bento et al. 2017; Ameline et al. 2021). Such epistasis may even result in apparently “forbidden” host resistotypes (Ameline et al. 2021). The complete absence of certain attachment site combinations among our isolates raises the possibility of “forbidden” infectotypes for the parasite as well (Supporting information Fig. S11B). Alternatively, the spatial structure inherent in our dataset may contribute to nonrandom phenotype distributions. Our current dataset does not allow us to distinguish between these alternatives.

### MULTIPLE WAYS TO INFECT A HOST

Our survey revealed that *P. ramosa* spores can attach not only to the foregut and hindgut, but also to various other sites, all of which are part of the cuticle that is regenerated when the *Daphnia* molts. These findings suggest that the cuticle is highly heterogeneous and may evolve in response to parasite selection. The molecules on the host cuticle to which the parasite spores attach are likely to be glycosylated proteins, as suggested by mapping of resistance genes in *D. magna* (Bento et al. 2017; Bourgeois et al. 2017, Bento et al. 2020). Bacterial attachment to the cuticle of nematodes has also been shown to be variable, but less so than the diversity described here (Davies 2009). This diversity in attachment sites modifies our understanding of stepwise infection processes, both for the *Daphnia–Pasteuria* system (Ebert et al. 2016; Hall et al. 2019; Hite 2020; Izhar et al. 2020) and more generally for host–parasite systems (Hall et al. 2017). The original model assumed that steps of infection follow a straight line from host–parasite encounter to host death. Here, we find that parasites follow the same path (host–parasite encounter and spore activation step) until the attachment step, when their paths diverge to use different entry points into the host, with attachment and penetration working in parallel at several sites. The following step, within‐host proliferation, likely proceeds similarly for parasites that entered through different sites (Bento et al. 2020). Other systems with multiple parasite entry sites are known (e.g. Raoult et al. 2005; Vizoso et al. 2005; Beyer and Turnbull 2009; Giordano et al. 2015), suggesting that a general model for host infection should include parallel steps.

While we found clear evidence that parasites can attach to several different sites on the host's cuticle, we could not verify that all these sites are entry routes into the host. Sites R (rectum), A (anus), and LA (all trunk limbs) are candidates for putative “dead‐end” attachment sites, though more sampling and testing is needed to better characterize these sites. If attachment sites exist that cannot lead to infection, such sites would be of no use to the parasite. The surface structure of these sites may resemble other sites that do lead to infection in the same or different host genotypes. In the host, there may be no selection on the genes underlying these sites, as they may not be detrimental with regard to infection. On the contrary, these sites may even benefit the host by sequestering parasite spores that would otherwise attach to a site where infection is possible. This hypothesis is analogous to the dilution effect (Keesing et al. 2006), which states that resistant hosts (here “resistant sites”) may reduce pathogen pressure on susceptible hosts (here: sites leading to infection), by reducing the number of transmission particles.

### CONCLUSIONS: IMPLICATIONS FOR RED QUEEN COEVOLUTION

High diversity in the *Pasteuria* parasite seems to match what is known from the *Daphnia* host, being consistent with a key prediction of the classical Red Queen model of coevolution, with both antagonists evolving under negative frequency‐dependent selection. Together with previous findings, it is becoming increasingly clear that *D. magna* resistance and *P. ramosa* infectivity are complex traits mediated by multiple loci with strong effects (Luijckx et al. 2013; Bento et al. 2020, 2017; Bourgeois et al. 2017; Andras et al. 2020; Ameline et al. 2021). How does this complexity relate to the Red Queen hypothesis of coevolution? In the earliest formulation of the matching allele model (which is assumed under the Red Queen Hypothesis), Clarke argues that increased genetic complexity would be *more likely* to maintain genetic diversity in coevolving host–parasite systems (Clarke 1976). However, since then it has been predicted that Red Queen allele dynamics will be strongest when resistance and infectivity are encoded by few genetic loci with strong effect and epistasis (Otto and Nuismer 2004; Brockhurst et al. 2014). The real number of loci for host resistance and parasite infectivity is not known in the *Daphnia–Pasteuria* system, but it is clear that there are multiple loci in both antagonists (Metzger et al. 2016b; Bento et al. 2017, Bento et al. 2020; Ameline et al. 2021; this study). Most attempts to map loci have used material from diverse populations, which would exaggerate diversity as expected in single populations. Still, our current study shows that diversity is also high within a single population, as has also been shown for the host (Ameline et al. 2021), possibly as a consequence of elevated effective migration rates for host resistance alleles and parasite infectivity alleles (Ebert and Fields 2020). However, genetic interactions among loci, as are known for resistance genes and seem likely for infectivity genes, strongly constrain the expression of phenotypes and, thus, impact the evolvability of the system. How this will influence Red Queen dynamics is not clear. In comparison with studies relying on only genotype data, which is the case for many coevolution studies including much of the work on MHC diversity (Leffler et al. 2013; Lenz et al. 2013; Robinson et al. 2017; Biedrzycka et al. 2018; Dudek et al. 2019), functional studies testing for the specific effects of the interaction phenotypes have more power to detect genetic mechanisms underlying host–parasite coevolution.

## AUTHOR CONTRIBUTIONS

Conceptualization: M.A.F. and D.E.; Methodology: M.A.F., C.A., B.H.; Resources: M.A.F., J.P.A. and D.E; Investigation: M.A.F. and M.K.; Formal analysis: M.A.F., P.D.F and D.E.; Software: M.A.F. and P.D.F; Writing—original draft: M.A.F.; Writing review and editing: M.A.F, C.A., M.K., B.H., P.D.F., J.P.A. and D.E.; and Funding acquisition: D.E.

Associate Editor: Prof. Mike Boots

Handling Editor: Prof. Tracey Chapman

## Supporting information


**Figure S1** Attachment of *Pasteuria ramosa* isolates in 12 genotypes of the *Daphnia magna* host to 8 attachment sites.

**Figure S2** Population heatmaps.

**Figure S3** Experimental setup for infection experiment

**Figure S4** Repeatability of attachment tests.

**Figure S5** Infectivity test attachment and infection results

**Figure S6** Distinguishing attachment phenotypes R, D and R/D.

**Figure S7** Density distributions of spore attachment at each described site

**Figure S8** Binary versions of the quantitative consensus matrices presented in figure 3.

**Figure S9** Total hosts infected plotted against total attachment sites for each of 51 *Pasteuria ramosa* isolates.

**Figure S10** Heatmaps showing site‐specific attachment diversity.

**Figure S11** Attachment site correlations.

Supplement Material

Supplemental table 1: Metadata for P. ramosa isolates and D. magna clones

Supplemental table 2: Attachment test raw data for full matrix (from figure S1)

Supplemental table 3: Loadings for principal component analysis
