# Supplementary material for: Infection phenotypes of a coevolving parasite are highly diverse, structured, and specific
Source: Evolution. 2021 Aug 30;75(10):2540–54. doi: 10.1111/evo.14323 (PMC9290032; doi:10.1111/evo.14323)
Supplement: Supplementary file 1 — Figure S1 Attachment of Pasteuria ramosa isolates in 12 genotypes of the Daphnia magna host to 8 attachment sites. [file EVO-75-2540-s011.pdf]

a.

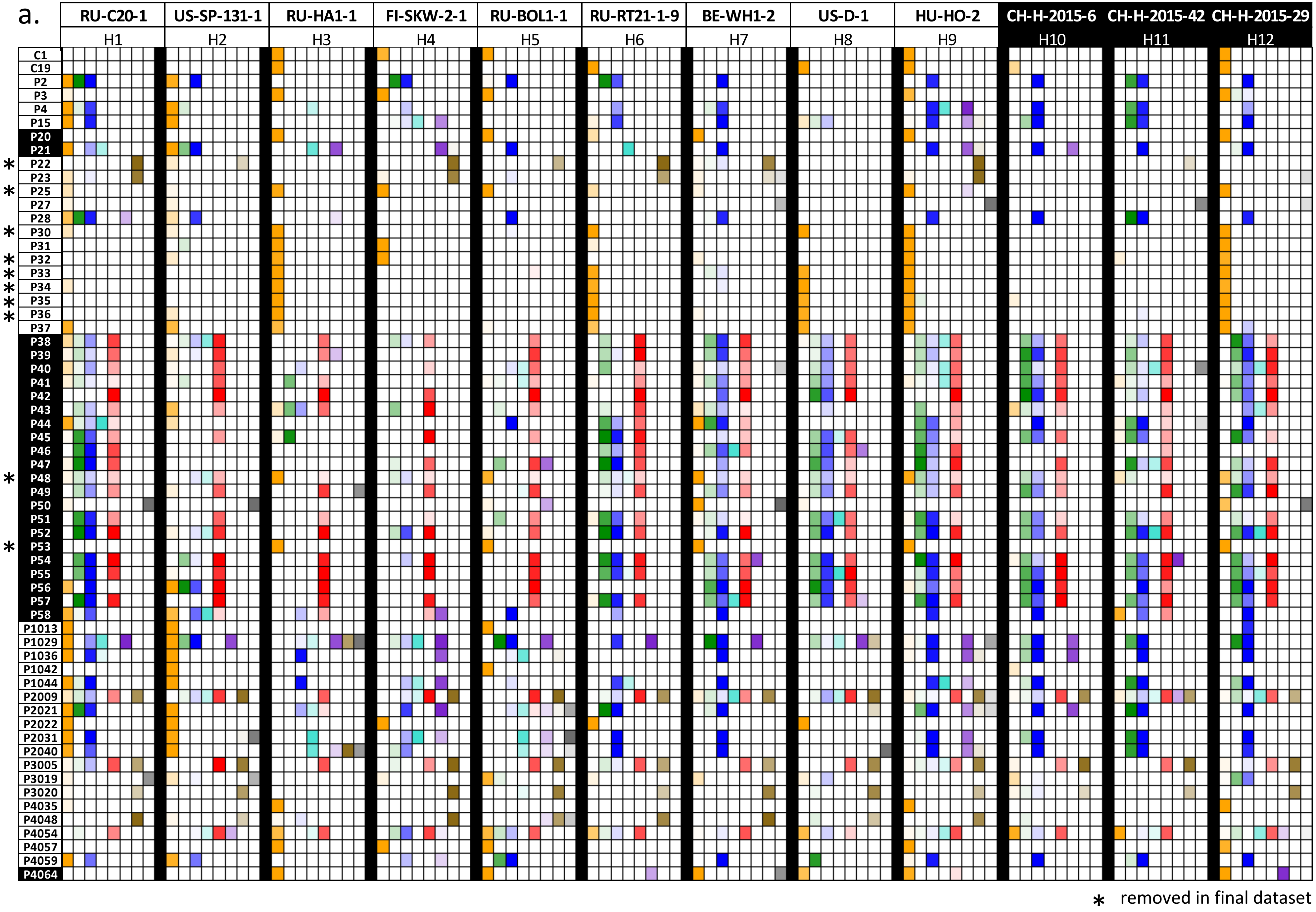

b.

	Site	count >0.5	percent
F	Foregut	137	18.7
R	Rectum *	66	9.0
D	Distal hindgut	171	23.4
A	Anus *	3	0.4
E	External postabdomen	156	21.3
L4	Trunk limb 4	22	3.0
L5	Trunk limb 5	34	4.6
LA	All trunk limbs *	1	0.1

* removed in final dataset
