# Supplementary figures and images for: Infection phenotypes of a coevolving parasite are highly diverse, structured, and specific
Source: Evolution. 2021 Aug 30;75(10):2540–54. doi: 10.1111/evo.14323 (PMC9290032; doi:10.1111/evo.14323)

# Attachment site / host clone combinations

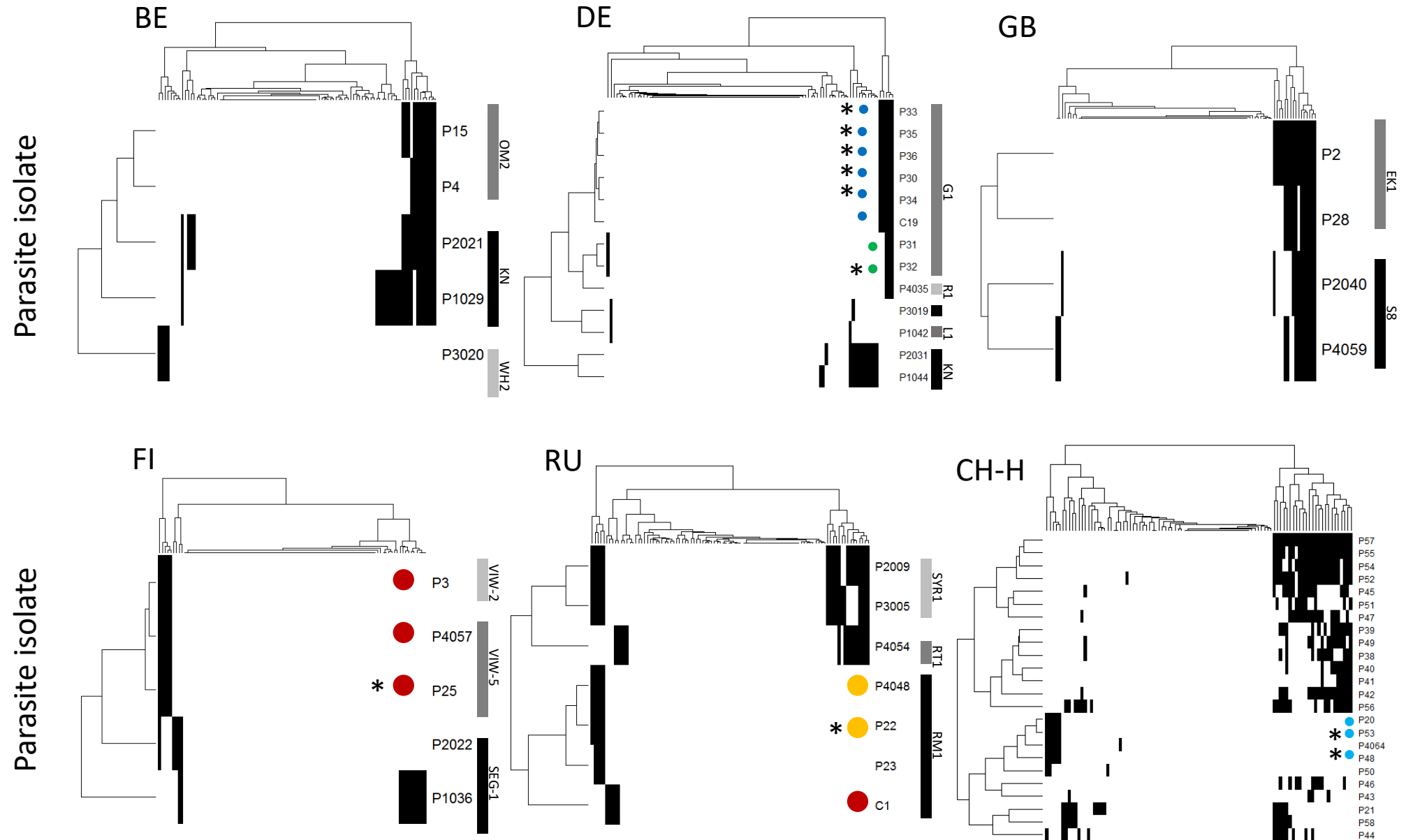

Supplement: Supplementary file 2 — Figure S2 Population heatmaps. [file EVO-75-2540-s009.pdf]

a.

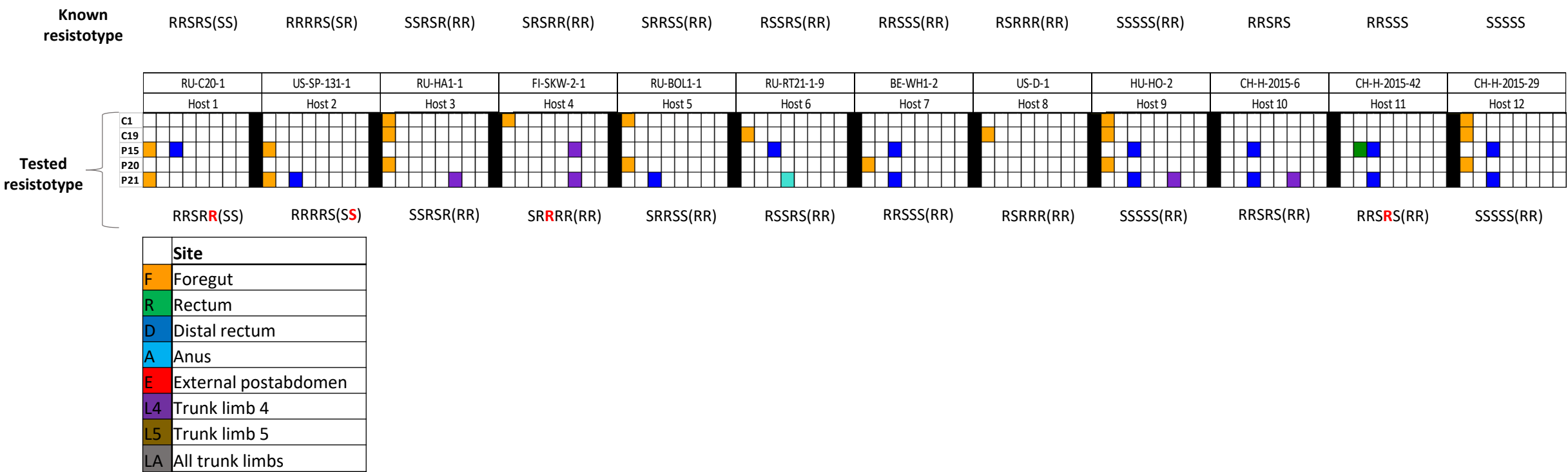

b.

| Percent variance |      |      |      |      |      |      |      |      |      |
|------------------|------|------|------|------|------|------|------|------|------|
| Effect           | all  | F    | D    | E    | L4   | L5   | R    | A    | LA   |
| host             | 6.1  | 0    | 14   | 0.04 | 0    | 2.9  | 12   | 0    | 0    |
| parasite         | 31.7 | 0    | 56   | 94.9 | 4.3  | 90.4 | 41.9 | 0    | 0    |
| host x parasite  | 47   | 97.4 | 17.9 | 1    | 91.8 | 2.2  | 23.7 | 90.7 | 94.2 |
| residual         | 15.3 | 2.6  | 12.1 | 4.1  | 3.9  | 4.4  | 22.4 | 9.3  | 5.8  |

Supplement: Supplementary file 4 — Figure S4 Repeatability of attachment tests. [file EVO-75-2540-s001.pdf]

a.

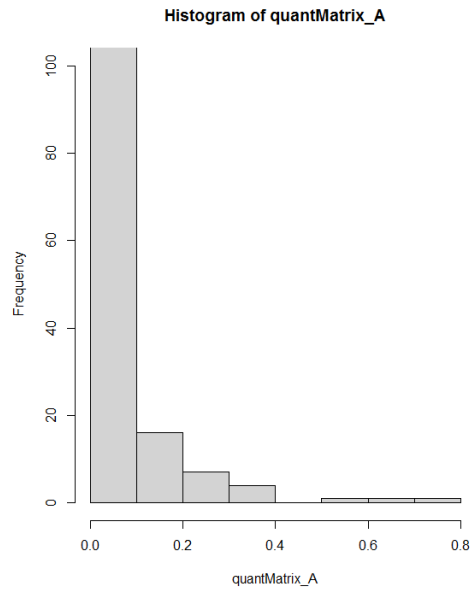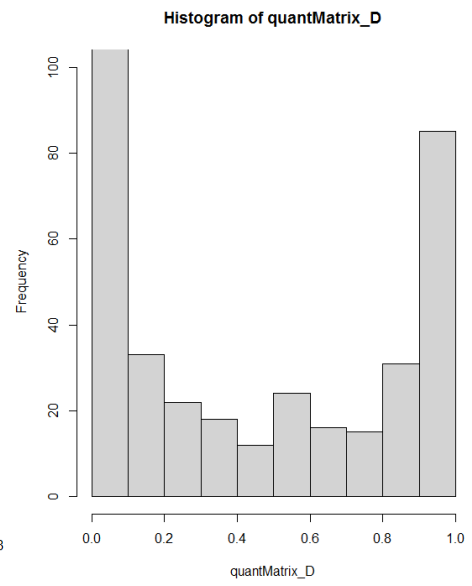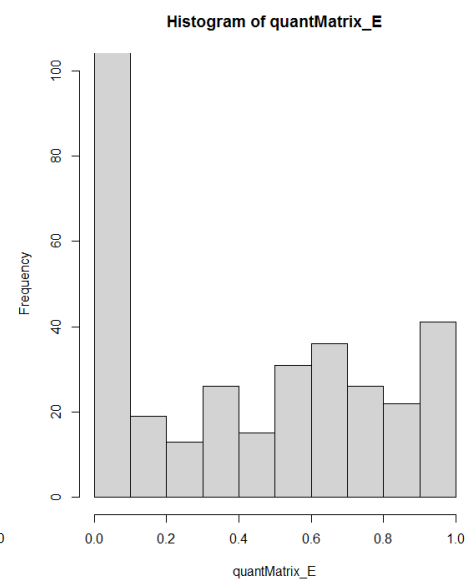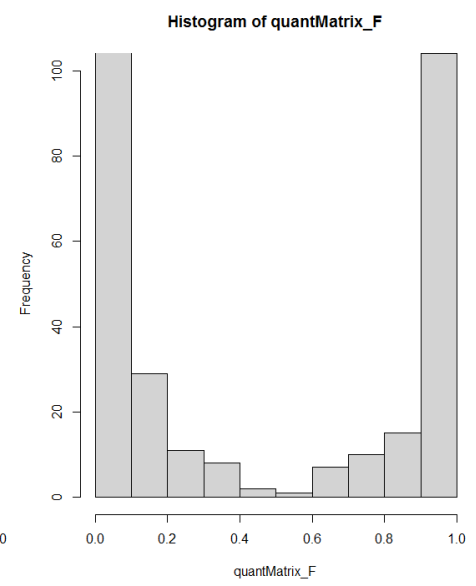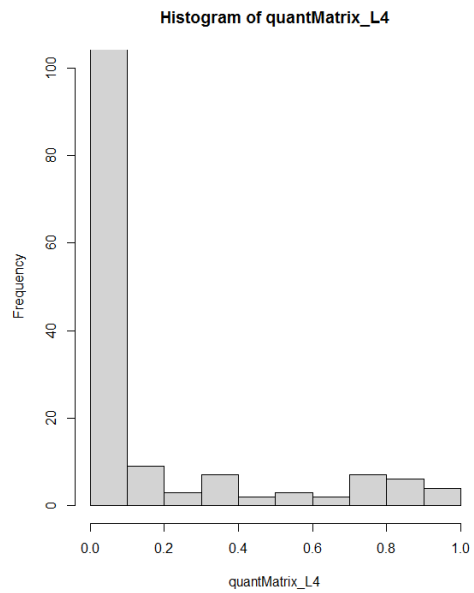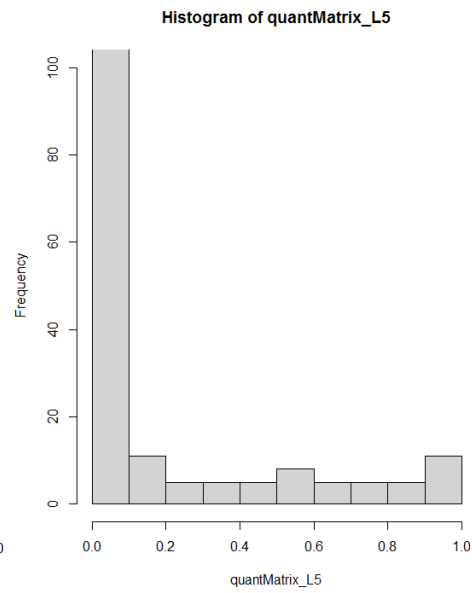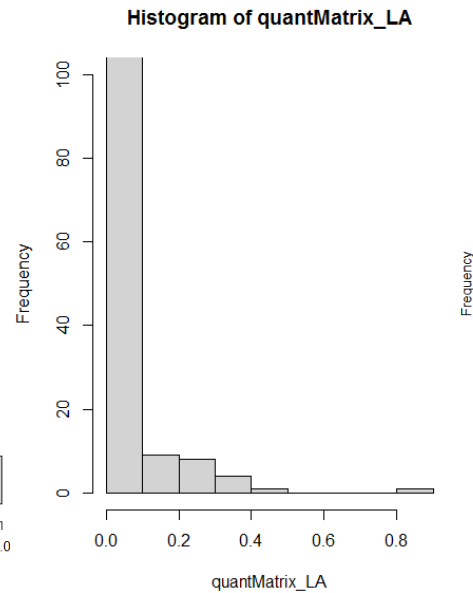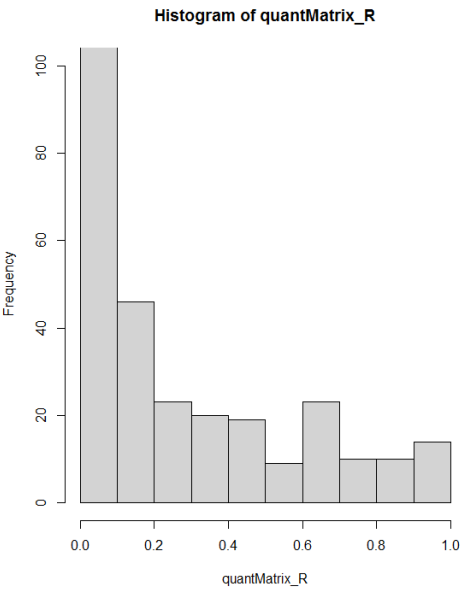

b.

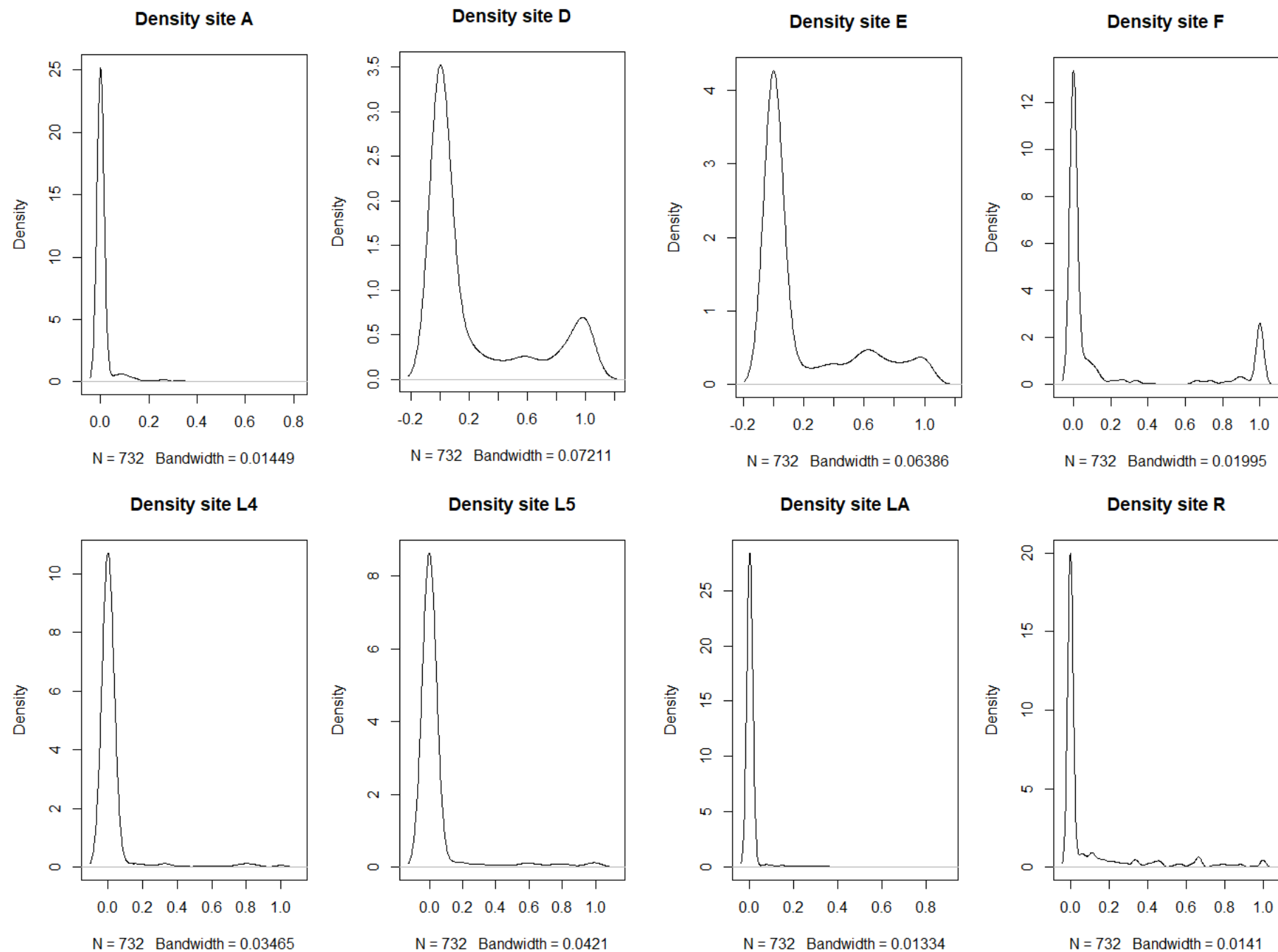

Supplement: Supplementary file 7 — Figure S7 Density distributions of spore attachment at each described site [file EVO-75-2540-s002.pdf]

a.

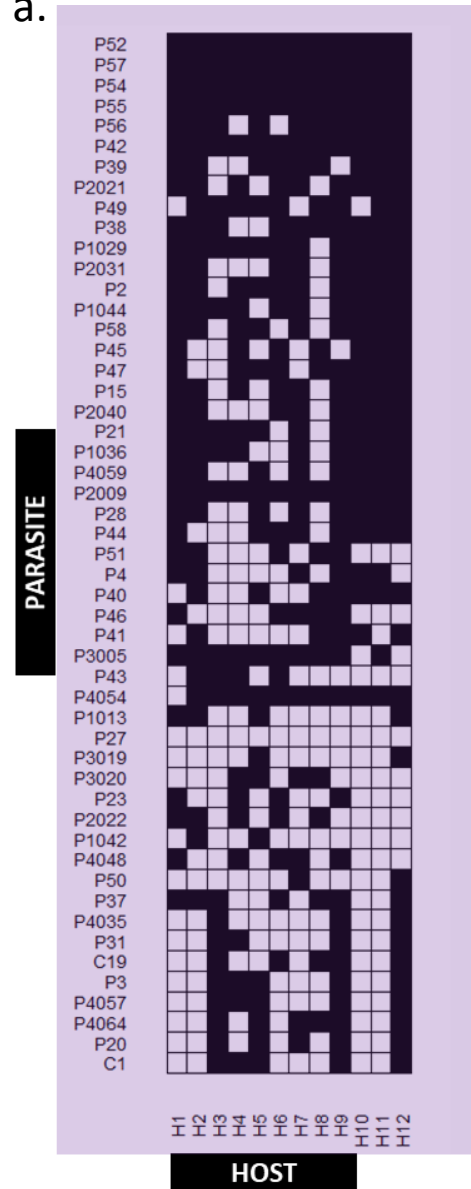

b.

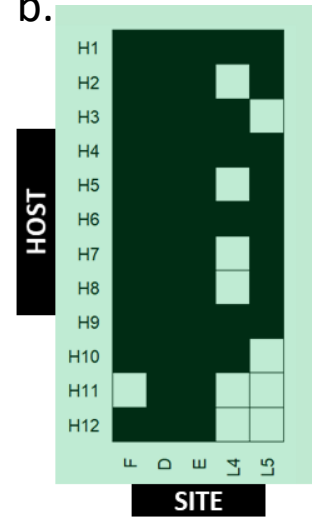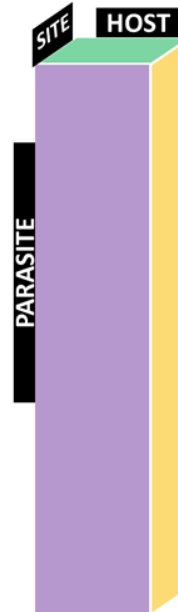

c.

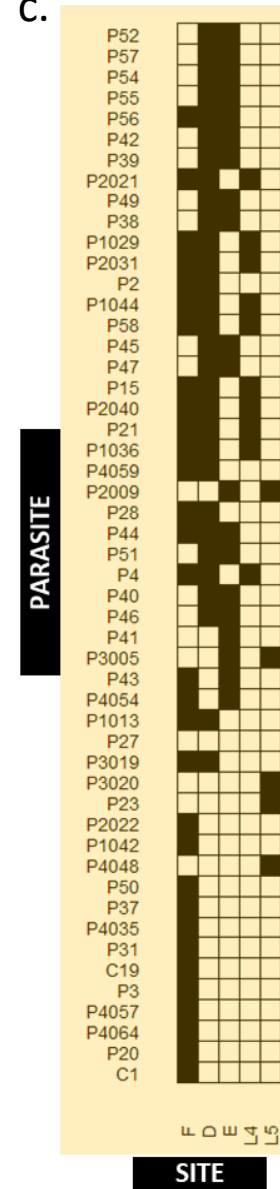

Supplement: Supplementary file 8 — Figure S8 Binary versions of the quantitative consensus matrices presented in figure 3. [file EVO-75-2540-s005.pdf]

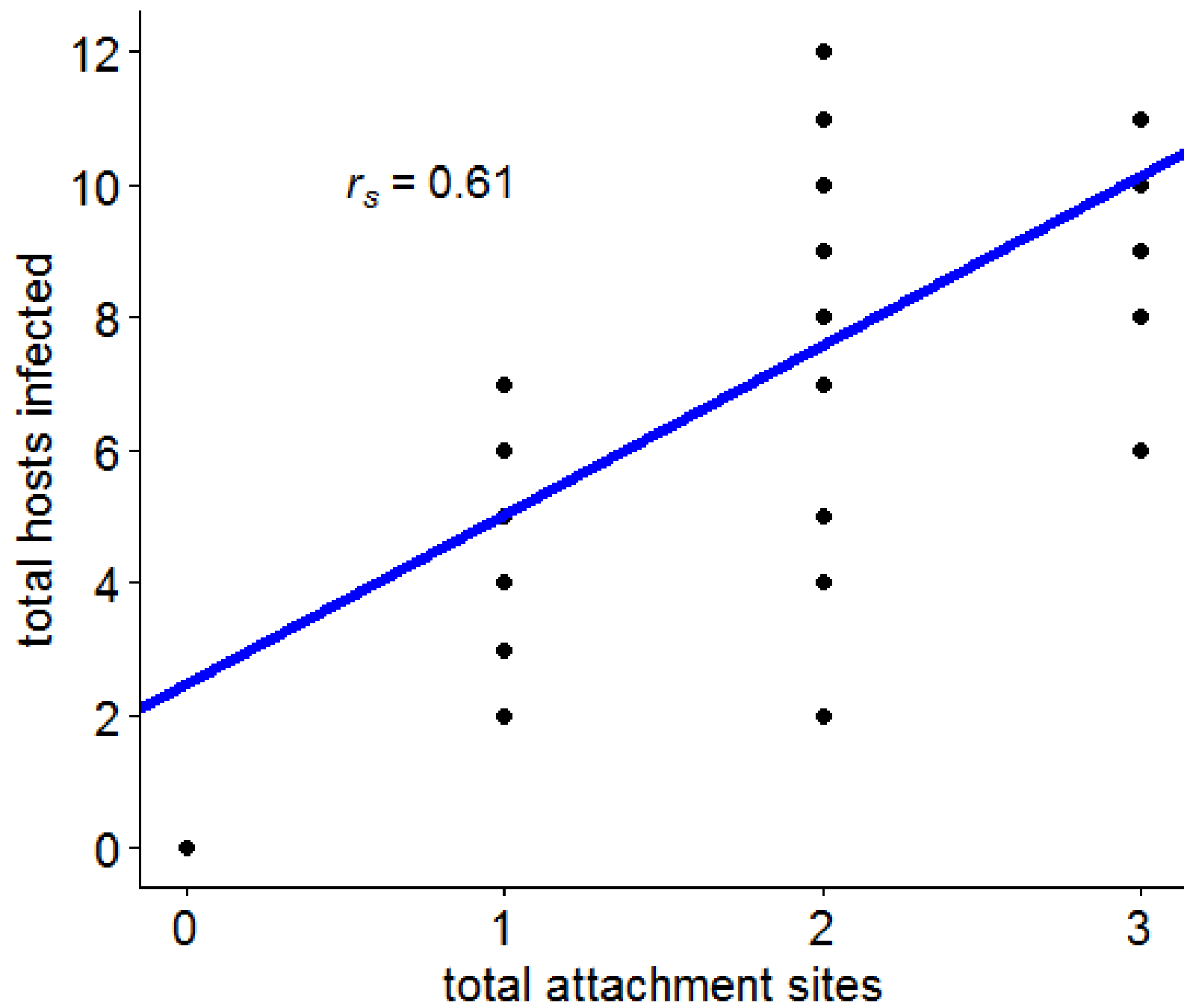

Supplement: Supplementary file 9 — Figure S9 Total hosts infected plotted against total attachment sites for each of 51 Pasteuria ramosa isolates. [file EVO-75-2540-s010.pdf]

a.

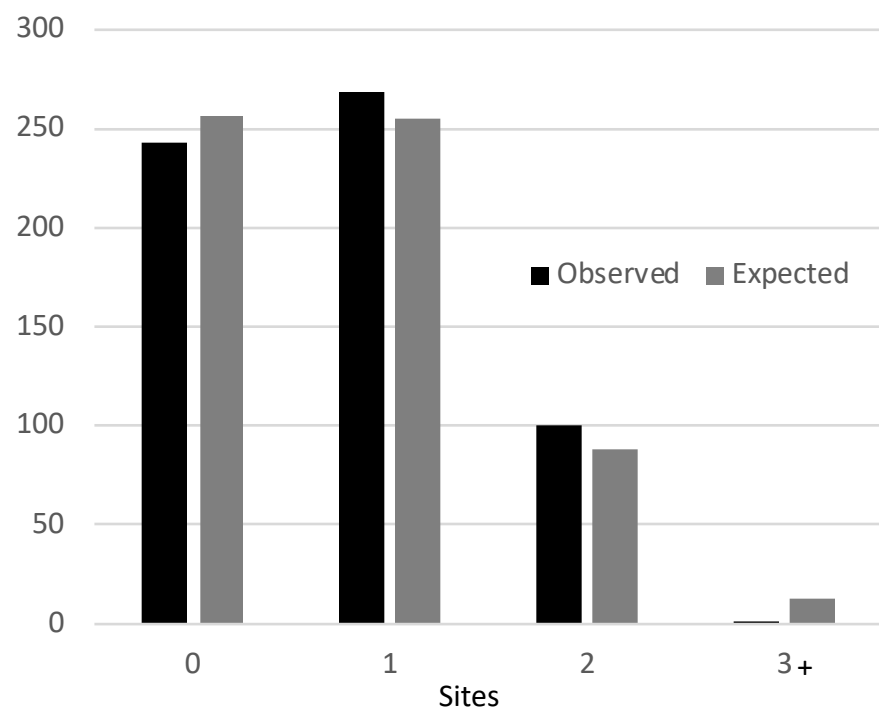

| Sites | Observed | Expected | Difference | Difference Sq. | Diff. Sq. / Exp Fr. |
|-------|----------|----------|------------|----------------|---------------------|
| 0     | 243      | 256      | -13.00     | 169.00         | 0.66                |
| 1     | 268      | 255      | 13.00      | 169.00         | 0.66                |
| 2     | 100      | 88       | 12.00      | 144.00         | 1.64                |
| 3+    | 1        | 13       | -12.00     | 144.00         | 11.08               |

 $\chi^2 = 14.036$ 
 $p = 0.00286$ 

b.

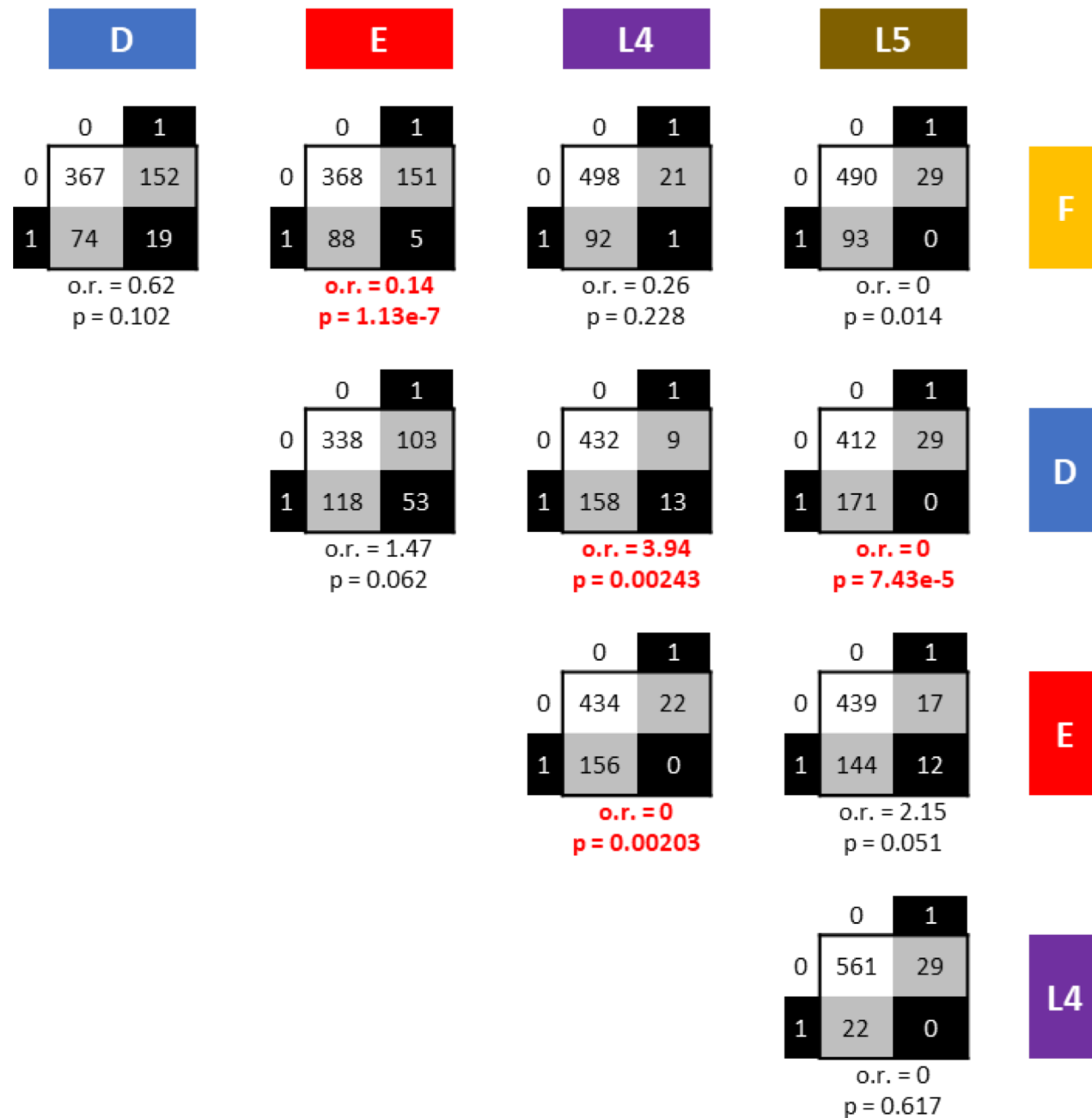

Supplement: Supplementary file 11 — Figure S11 Attachment site correlations. [file EVO-75-2540-s006.pdf]
