# Supplementary material for: Infection phenotypes of a coevolving parasite are highly diverse, structured, and specific
Source: Evolution. 2021 Aug 30;75(10):2540–54. doi: 10.1111/evo.14323 (PMC9290032; doi:10.1111/evo.14323)
Supplement: Supplementary file 3 — Figure S3 Experimental setup for infection experiment [file EVO-75-2540-s007.pdf]

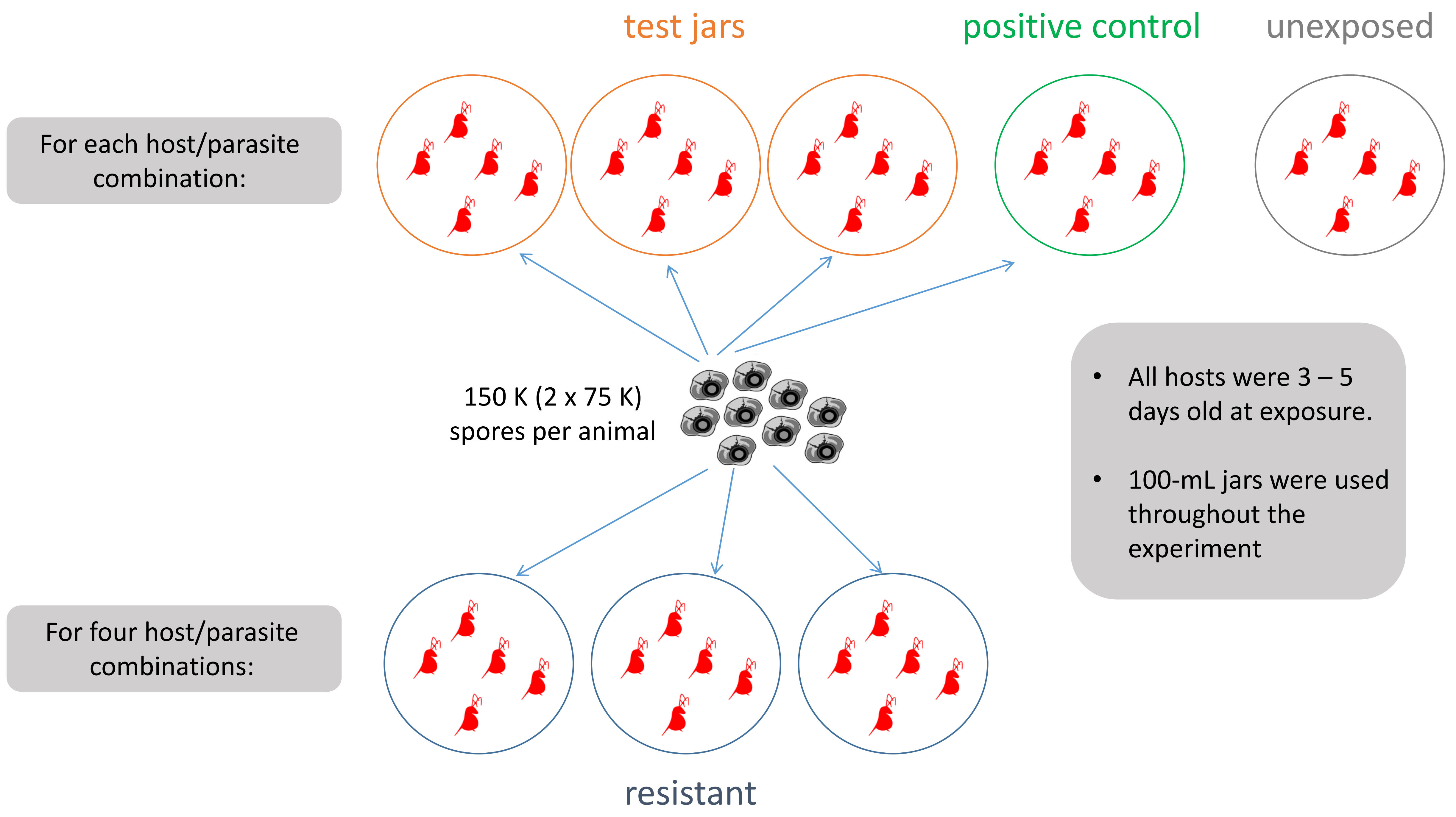
test jars
positive control
unexposed
For each host/parasite combination:
150 K (2 x 75 K) spores per animal
All hosts were 3 – 5 days old at exposure.
100-mL jars were used throughout the experiment
For four host/parasite combinations:
resistant
