# Supplementary material for: Infection phenotypes of a coevolving parasite are highly diverse, structured, and specific
Source: Evolution. 2021 Aug 30;75(10):2540–54. doi: 10.1111/evo.14323 (PMC9290032; doi:10.1111/evo.14323)
Supplement: Supplementary file 5 — Figure S5 Infectivity test attachment and infection results [file EVO-75-2540-s014.pdf]

a. Test

treatment	attach_site	parasite_isolate	host_clone	prop_infect	D	A	R	F	E	L4	L5	LA
test	F	P2031	US-SP131-1	0.87	0.00	0.00	0.00	0.95	0.00	0.00	0.05	0.30
test	F	P4064	BE-WH1-2	0.93	0.00	0.00	0.00	1.00	0.00	0.00	0.00	0.36
test	D	P21	CH-H-2015-29	0.73	1.00	0.00	0.06	0.00	0.00	0.00	0.00	0.00
test	D	P44	CH-H-2015-42	1.00	0.96	0.00	0.63	0.04	0.17	0.00	0.00	0.04
test	E	P38	RU-RT21-1-9	0.47	0.00	0.00	0.25	0.00	0.90	0.00	0.00	0.00
test	E	P38	US-SP131-1	0.40	0.22	0.19	0.07	0.04	0.89	0.00	0.00	0.00
test	E	P40	CH-H-2015-42	0.87	0.10	0.20	0.25	0.00	0.65	0.00	0.00	0.14
test	E	P4054	BE-WH1-2	0.00	0.07	0.00	0.00	0.04	0.67	0.00	0.00	0.00
test	E	P4054	CH-H-2015-29	0.33	0.00	0.08	0.12	0.04	0.60	0.08	0.00	0.08
test	E	P4054	US-SP131-1	0.47	0.04	0.08	0.00	0.00	0.77	0.07	0.00	0.00
test	E	P41	HU-HO-2	0.93	0.04	0.07	0.04	0.11	0.56	0.00	0.00	0.00
test	E	P41	US-D-1	0.80	0.40	0.00	0.28	0.00	0.60	0.00	0.00	0.00
test	E	P43	RU-RT21-1-9	0.13	0.00	0.00	0.12	0.00	0.71	0.00	0.00	0.00
test	E	P49	RU-BOL1-1	0.20	0.00	0.04	0.00	0.00	0.74	0.00	0.00	0.00
test	E	P49	RU-HA1-1	0.00	0.00	0.00	0.00	0.00	0.77	0.00	0.00	0.07
test	E	P57	FI-SKW-2-1	1.00	0.00	0.00	0.00	0.00	0.88	0.00	0.00	0.00
test	L4	P1029	RU-HA1-1	0.00	0.05	0.09	0.00	0.00	0.00	0.82	0.05	0.09
test	L4	P1036	FI-SKW-2-1	0.33	0.00	0.00	0.00	0.00	0.00	0.78	0.00	0.00
test	L4	P1044	FI-SKW-2-1	0.67	0.19	0.13	0.00	0.00	0.00	0.88	0.00	0.00
test	L4	P15	FI-SKW-2-1	0.20	0.08	0.14	0.03	0.00	0.00	0.54	0.00	0.00
test	L4	P21	FI-SKW-2-1	0.27	0.00	0.00	0.00	0.00	0.00	0.88	0.06	0.00
test	L4	P21	RU-HA1-1	0.07	0.00	0.27	0.00	0.00	0.00	0.80	0.00	0.00
test	L5	P2009	RU-C20-1	0.80	0.28	0.00	0.16	0.25	0.47	0.00	0.72	0.00
test	L5	P22	FI-SKW-2-1	0.93	0.00	0.00	0.00	0.00	0.00	0.00	0.94	0.00
test	L5	P22	RU-RT21-1-9	0.67	0.00	0.00	0.00	0.00	0.00	0.00	1.00	0.00
test	L5	P23	BE-WH1-2	0.80	0.00	0.00	0.00	0.09	0.00	0.00	0.22	0.10
test	L5	P3005	FI-SKW-2-1	0.73	0.00	0.00	0.07	0.00	0.40	0.00	1.00	0.00
test	L5	P3020	RU-BOL1-1	0.80	0.00	0.04	0.00	0.00	0.00	0.00	0.58	0.00
test	L5	P3020	US-D-1	0.47	0.00	0.00	0.05	0.00	0.00	0.00	0.55	0.00
test	L5	P3020	US-SP131-1	0.07	0.00	0.00	0.00	0.00	0.00	0.00	0.45	0.00
test	L5	P4048	RU-BOL1-1	0.80	0.00	0.00	0.00	0.00	0.00	0.07	0.45	0.14
test	L5	P4048	RU-C20-1	0.47	0.00	0.00	0.00	0.00	0.00	0.00	1.00	0.00

b. Positive control

parasite_isolate	host_clone	prop_infect	F	R	D	A	E	L4	L5	LA
P1029	HU-HO-2	0.80	0.09	0.04	0.96	0.00	0.00	0.83	0.00	0.17
P1036	HU-HO-2	1.00	0.08	0.00	0.92	0.00	0.00	0.77	0.15	0.00
P1044	HU-HO-2	1.00	0.00	0.00	0.87	0.13	0.00	0.33	0.00	0.00
P15	HU-HO-2	1.00	0.05	0.00	0.86	0.00	0.00	0.45	0.09	0.00
P2009	HU-HO-2	1.00	0.10	0.03	0.03	0.00	0.66	0.00	0.79	0.06
P2021	HU-HO-2	0.80	0.00	0.44	0.97	0.00	0.00	0.54	0.16	0.08
P2031	HU-HO-2	1.00	0.00	0.05	1.00	0.00	0.00	0.68	0.00	0.00
P2040	HU-HO-2	1.00	0.00	0.00	1.00	0.00	0.00	0.59	0.18	0.00
P21	HU-HO-2	1.00	0.00	0.00	0.93	0.00	0.00	0.73	0.13	0.00
P22	HU-HO-2	1.00	0.00	0.00	0.00	0.00	0.00	0.00	1.00	0.00
P23	HU-HO-2	0.80	0.09	0.00	0.00	0.00	0.00	0.00	0.96	0.00
P3005	HU-HO-2	1.00	0.03	0.06	0.06	0.03	0.45	0.00	0.82	0.00
P3020	DE-K35-Iinb1	1.00	0.07	0.00	0.00	0.00	0.00	0.00	0.80	0.00
P38	t1_10.3_2i_2	0.20	0.08	0.65	0.81	0.04	0.58	0.00	0.00	0.00
P40	t1_10.3_2i_6	0.80	0.04	0.88	0.79	0.00	0.83	0.00	0.00	0.00
P4048	HU-HO-2	1.00	0.00	0.00	0.00	0.00	0.00	0.00	0.74	0.00
P4054	HU-HO-2	1.00	0.06	0.03	0.06	0.06	0.68	0.00	0.00	0.00
P4064	HU-HO-2	0.80	0.89	0.00	0.00	0.00	0.11	0.00	0.00	0.00
P41	t1_10.3_2i_11	0.60	0.00	0.48	0.80	0.00	0.44	0.00	0.00	0.00
P43	t1_10.3_2	0.00	0.00	0.72	0.68	0.04	0.64	0.00	0.00	0.00
P44	PL-W2-1	0.40	0.92	0.00	0.08	0.15	0.15	0.00	0.00	0.00
P47	DE-G1-106-C	0.00	0.00	0.47	0.71	0.00	0.53	0.00	0.00	0.00
P49	US-D-1	0.80	0.03	0.18	0.43	0.00	0.63	0.00	0.00	0.00
P57	GB-EP-1-C	0.40	0.09	0.68	0.65	0.09	0.88	0.00	0.00	0.00

c. Resistant

treatment	attach_site	parasite_isolate	host_clone	prop_infect	D	A	R	F	E	L4	L5	LA
resistant		P1036	RU-RT21-1-9	0.13	0.00	0.00	0.00	0.00	0.00	0.00	0.00	0.00
resistant		P2031	US-D-1	0.33	0.00	0.00	0.00	0.00	0.00	0.00	0.00	0.00
resistant		P21	US-D-1	0.13	0.00	0.00	0.00	0.00	0.00	0.00	0.00	0.00
resistant		P4064	US-SP131-1	0.00	0.00	0.00	0.00	0.00	0.00	0.00	0.00	0.00

0.01 – 0.5

> 0.5
