# Supplementary material for: Infection phenotypes of a coevolving parasite are highly diverse, structured, and specific
Source: Evolution. 2021 Aug 30;75(10):2540–54. doi: 10.1111/evo.14323 (PMC9290032; doi:10.1111/evo.14323)
Supplement: Supplementary file 6 — Figure S6 Distinguishing attachment phenotypes R, D and R/D. [file EVO-75-2540-s004.pdf]

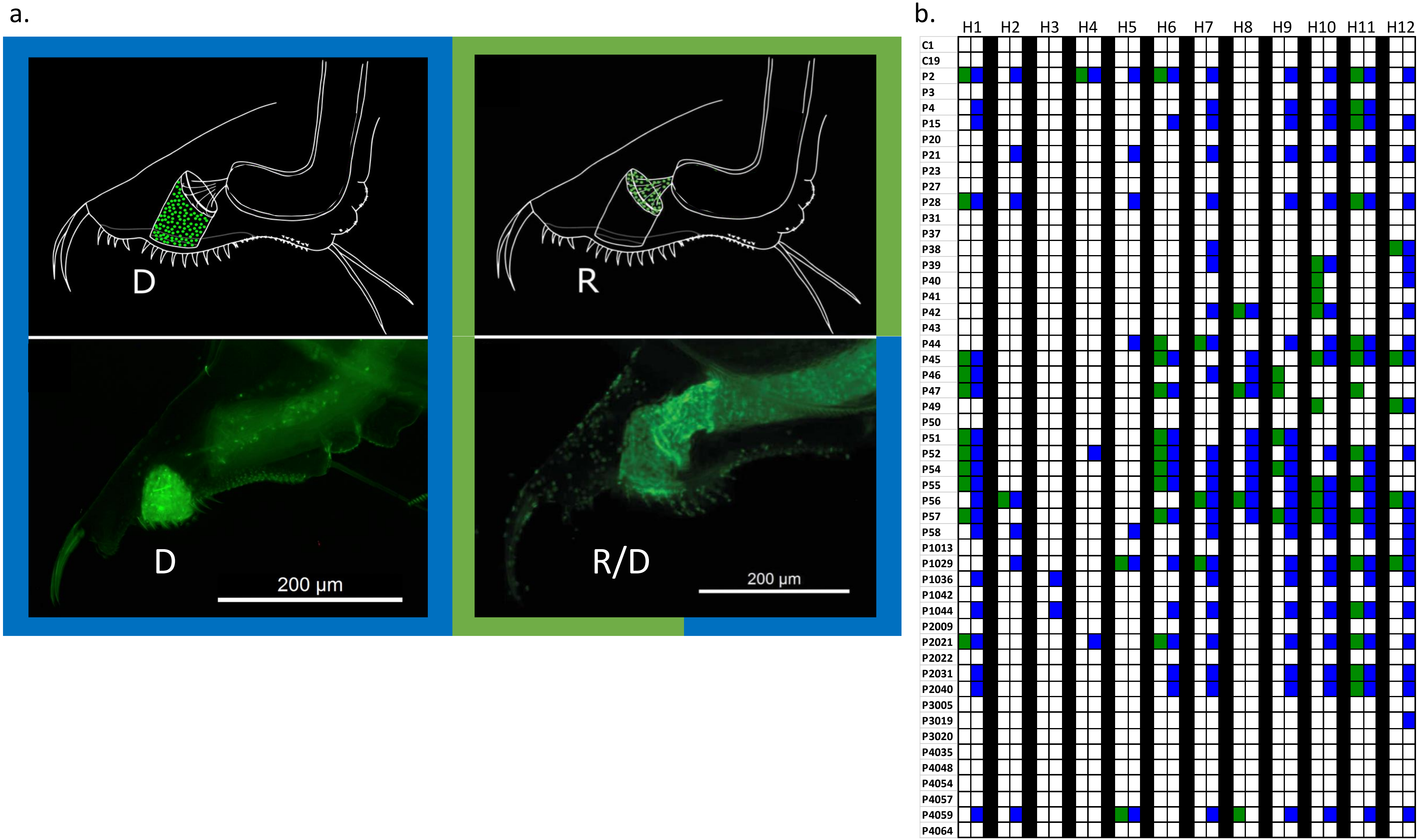
a.
D
R
D
R/D
200 µm
200 µm
b.
H1 H2 H3 H4 H5 H6 H7 H8 H9 H10 H11 H12
C1
C19
P2
P3
P4
P15
P20
P21
P23
P27
P28
P31
P37
P38
P39
P40
P41
P42
P43
P44
P45
P46
P47
P49
P50
P51
P52
P54
P55
P56
P57
P58
P1013
P1029
P1036
P1042
P1044
P2009
P2021
P2022
P2031
P2040
P3005
P3019
P3020
P4035
P4048
P4054
P4057
P4059
P4064
