# Supplementary material for: Infection phenotypes of a coevolving parasite are highly diverse, structured, and specific
Source: Evolution. 2021 Aug 30;75(10):2540–54. doi: 10.1111/evo.14323 (PMC9290032; doi:10.1111/evo.14323)
Supplement: Supplementary file 10 — Figure S10 Heatmaps showing site‐specific attachment diversity. [file EVO-75-2540-s015.pdf]

a.

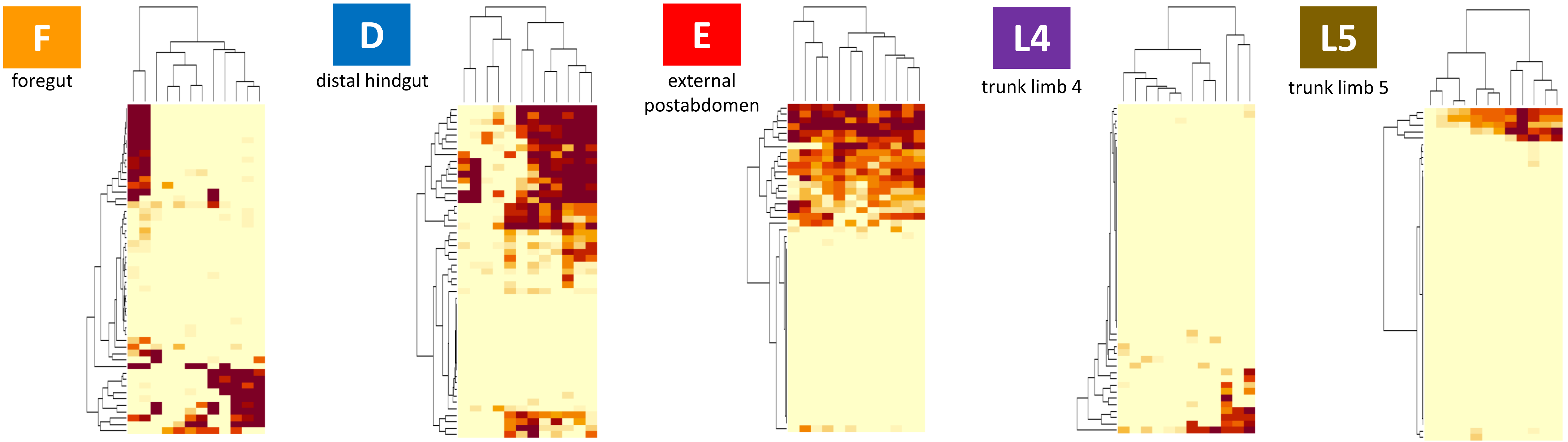
F
foregut
D
distal hindgut
E
external
postabdomen
L4
trunk limb 4
L5
trunk limb 5

b.

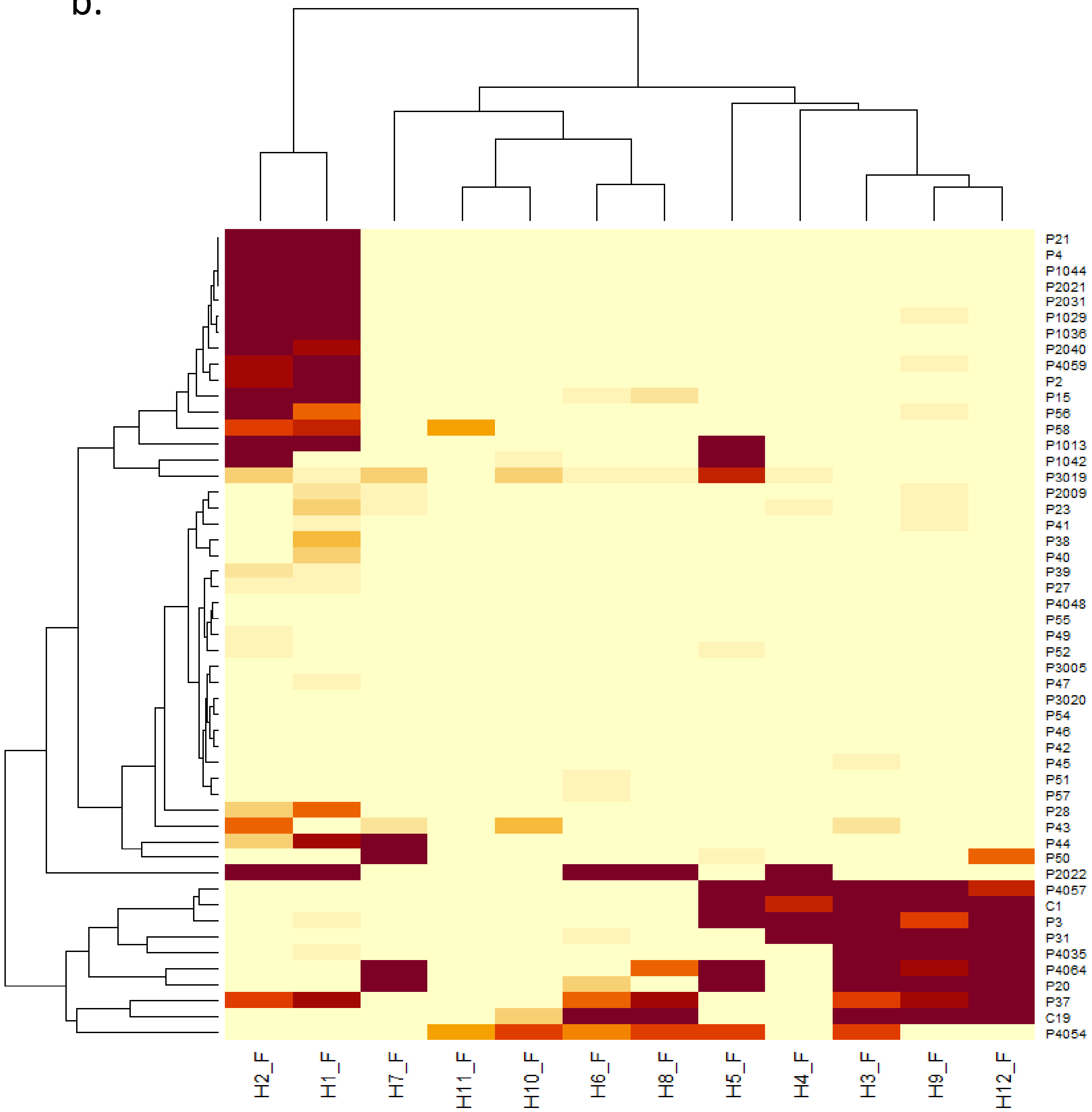
P21
P4
P1044
P2021
P2031
P1029
P1036
P2040
P4059
P2
P15
P56
P58
P1013
P1042
P3019
P2009
P23
P41
P38
P40
P39
P27
P4048
P55
P49
P52
P3005
P47
P3020
P54
P46
P42
P45
P51
P57
P28
P43
P44
P50
P2022
P4057
C1
P3
P31
P4035
P4064
P20
P37
C19
P4054
H2_F
H1_F
H7_F
H11_F
H10_F
H6_F
H8_F
H5_F
H4_F
H3_F
H9_F
H12_F

c.

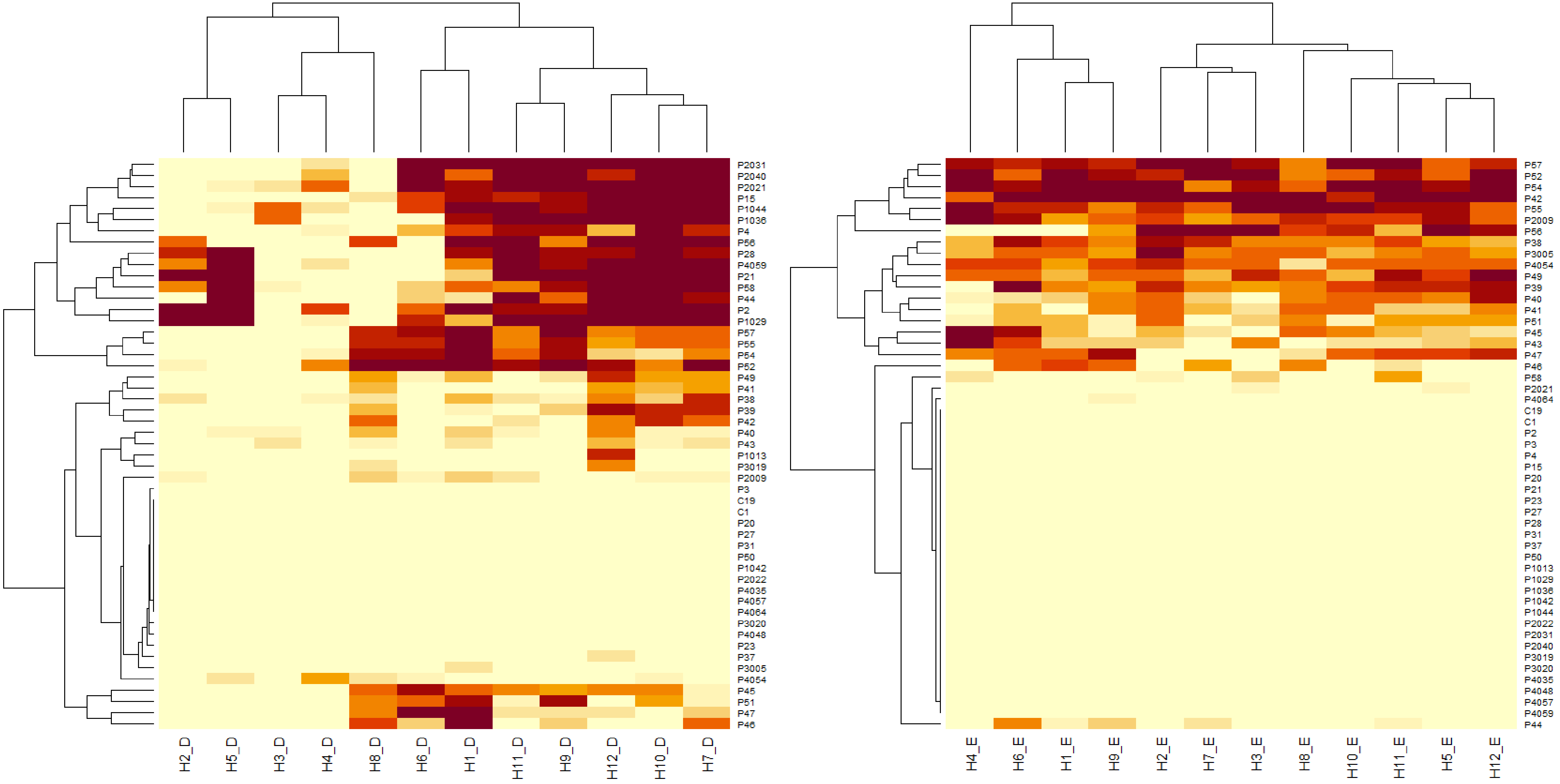
P2031
P2040
P2021
P15
P1044
P1036
P4
P56
P28
P4059
P21
P58
P44
P2
P1029
P57
P55
P54
P52
P49
P41
P38
P39
P42
P40
P43
P1013
P3019
P2009
P3
C19
C1
P20
P27
P31
P50
P1042
P2022
P4035
P4057
P4064
P3020
P4048
P23
P37
P3005
P4054
P45
P51
P47
P46
H2_D
H5_D
H3_D
H4_D
H8_D
H6_D
H1_D
H11_D
H9_D
H12_D
H10_D
H7_D
P57
P52
P54
P42
P55
P2009
P56
P38
P3005
P4054
P49
P39
P40
P41
P51
P45
P43
P47
P46
P58
P2021
P4064
C19
C1
P2
P3
P4
P15
P20
P21
P23
P27
P28
P31
P37
P50
P1013
P1029
P1036
P1042
P1044
P2022
P2031
P2040
P3019
P3020
P4035
P4048
P4057
P4059
P44
H4_E
H6_E
H1_E
H9_E
H2_E
H7_E
H3_E
H8_E
H10_E
H11_E
H5_E
H12_E

d.

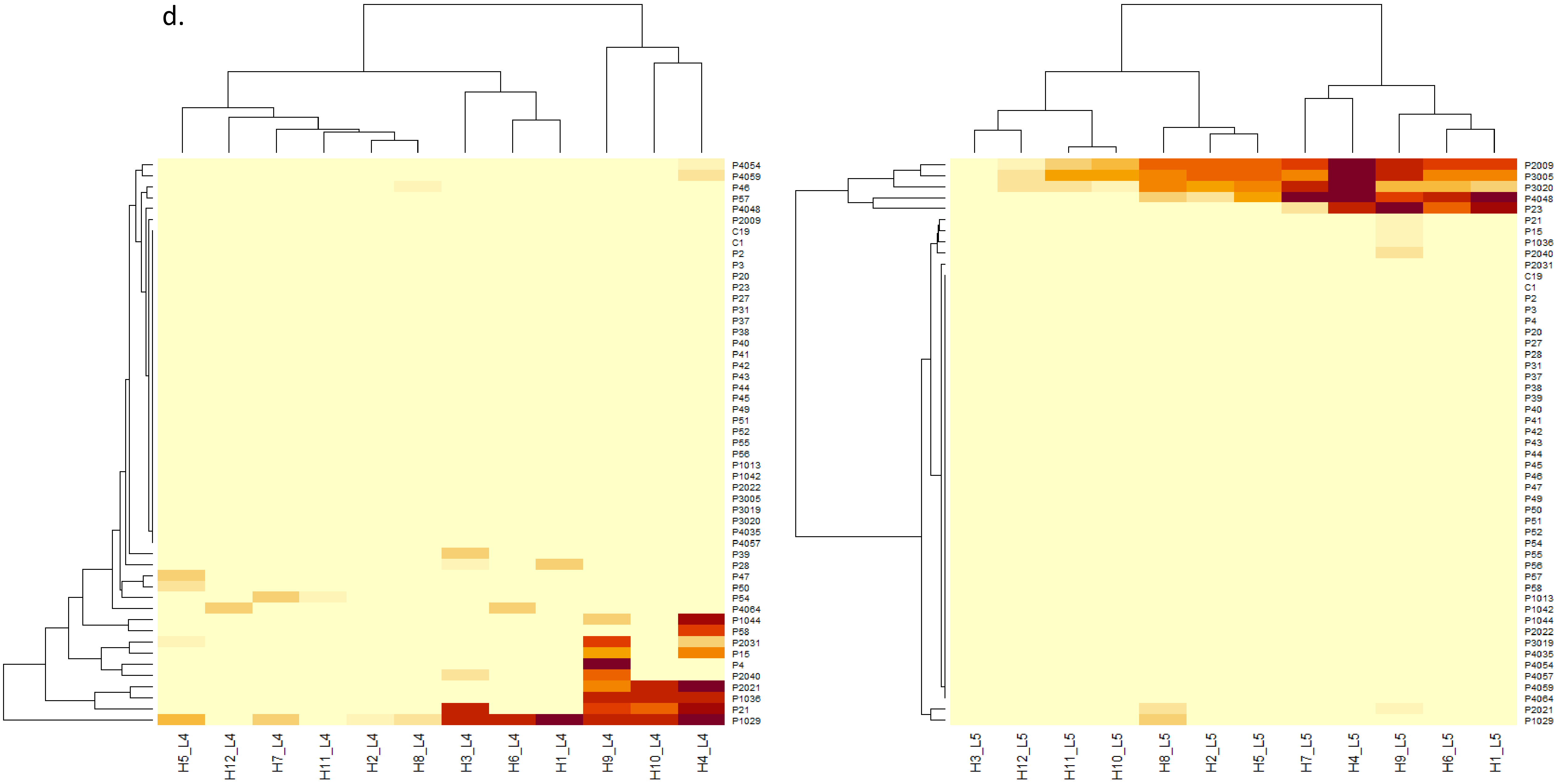
P4054
P4059
P46
P57
P4048
P2009
C19
C1
P2
P3
P20
P23
P27
P31
P37
P38
P40
P41
P42
P43
P44
P45
P49
P51
P52
P55
P56
P1013
P1042
P2022
P3005
P3019
P3020
P4035
P4057
P39
P28
P47
P50
P54
P4064
P1044
P58
P2031
P15
P4
P2040
P2021
P1036
P21
P1029
H5_L4
H12_L4
H7_L4
H11_L4
H2_L4
H8_L4
H3_L4
H6_L4
H1_L4
H9_L4
H10_L4
H4_L4
P2009
P3005
P3020
P4048
P23
P21
P15
P1036
P2040
P2031
C19
C1
P2
P3
P4
P20
P27
P28
P31
P37
P38
P39
P40
P41
P42
P43
P44
P45
P46
P47
P49
P50
P51
P52
P54
P55
P56
P57
P58
P1013
P1042
P1044
P2022
P3019
P4035
P4054
P4057
P4059
P4064
P2021
P1029
H3_L5
H12_L5
H11_L5
H10_L5
H8_L5
H2_L5
H5_L5
H7_L5
H4_L5
H9_L5
H6_L5
H1_L5
