# Supplementary material for: Infection phenotypes of a coevolving parasite are highly diverse, structured, and specific
Source: Evolution. 2021 Aug 30;75(10):2540–54. doi: 10.1111/evo.14323 (PMC9290032; doi:10.1111/evo.14323)
Supplement: Supplementary file 12 — Supplement Material [file EVO-75-2540-s013.pdf]

Supplementary methods and results: attachment sites

Attachment pictures

To describe the new attachment sites observed, we developed a protocol to take good quality images of the attachment. We infected small individuals (roughly 1 mm length) with fluorescently labelled spores in 96-well plates as described in (Duneau et al. 2011), using varying amounts of spores. In the attachment test, depending on the bacterial isolate, we routinely feed one individual 4000-10000 spores. The spore solutions used in the standard attachment test are created from five to ten homogenized infected *D. magna* individuals and diluted ten times (1:10 dilution). To avoid too strong fluorescence that would impede taking clear pictures, we here used 500-2000 spores of 1:10 dilutions or 500-10000 *P. ramosa* spores of 1:100 dilutions. The attachment test is performed using alive *D. magna*. To get clear pictures of the attachment, we however needed to immobilize the individuals. After the incubation period (about 30 min), we removed the medium and added 150 µL of 18% EtOH solution, in which individuals were left for 10 min. Individuals were then placed with a drop of solution between glass slide and cover slip separated by small pieces of playdough at the corners of the cover slip. We then observed and took images of the sample under a fluorescent lens microscope, within 10 minutes to avoid autofluorescence caused by the decomposition of the tissues. Unless mentioned otherwise, we used a Leica DM6 B microscope, objective 10x with 1x or 1.6x magnification, FluoCube: GFP. Images were produced using a Leica DFC7000 T camera connected to the microscope, using the program LAS v. 4.12 and the package “montage”, with a Z-step size of 2 µm (30-50 pictures per image). Details on the host and parasite genotypes, number of spores and dilutions used to produce the attachment images are given in Tables 1 and 2.

Complex attachment in the postabdomen

Attachment of *Pasteuria ramosa* spores has been observed in *Daphnia magna* until now on two parts of the gut wall, in the foregut (“F” attachment) and in the hindgut (Figs. 1, 2 and 3) (Duneau et al. 2011; Bento et al. 2020). However, the anatomy of the hindgut is complex and attachment in this region has not yet been characterized precisely. As newly isolated *P. ramosa* were used, new attachment sites were observed in the postabdomen region of the host (Fig. 2). First, we observed that attachment could occur in different parts of the hindgut, namely the distal hindgut (“D” attachment) and the rectum (“R” attachment) (Figs. 4 and 5). The rectum is a flexible membrane that links the distal end of the midgut to the seemingly harder distal hindgut (Fig. 2), producing variable observable patterns depending on the degree of extension of the membrane (compare Fig. 5B and C). Because of this, the rectum attachment can result difficult to score. For example, Figure 5D shows a possible artefact created by the mobility of the outer membrane of the midgut that looks like a rectum attachment but is probably not one. Attachment to both the distal part of the hindgut and the rectum is often observed, but it has not been quantified yet. Attachment in the distal part of the hindgut has been observed before with the P15 and P21 *P.ramosa* isolates, and the attachment pattern is clear, covering the whole cavity of the distal part of the hindgut (Fig. 4B and C), although attachment intensity can vary. As we tested new *P.ramosa* isolates, we observed much more variable attachment in the distal hindgut. Figures 4D to G show different patterns of partial attachment in the distal hindgut observed consistently across host and parasite genotypes.

Second, we observed that spores attached to the outer part of the postabdomen. We describe two further attachment sites, the anus “A” (Fig. 6) and the external abdomen “E” (Fig. 7) attachments.

The anus attachment describes spores attaching to the opening of the distal hindgut and on the larger postabdominal comb, while the external abdomen attachment describes attachment of spores on the entire surface of the postabdomen. These two attachments were observed separately (only “A”: Fig. 6B to E, only “E”: Fig. 7B and C) or together (Fig. 7D), although the intensity of each can vary. For example, both are observed in Figure 7D at the relative same intensity, but Figure 6F shows a more intense attachment to the anus than to the external abdomen. Figure 6F also shows that the anus attachment can be difficult to disentangle from a partial attachment in the distal hindgut.

Complex attachment on the appendages

Using new *P.ramosa* isolates further revealed a new attachment site, this time on one of the appendages, or trunk limbs, of the host. This new attachment on trunk limb 5 “L5” shows a clear pattern (Fig. 8). Additionally, several isolates showed attachment to the exopodic setae of trunk limb 4 “L4” (Fig. 9), and others showed attachment to all trunk limbs “LA” (Fig. 10).

The all-trunk-limbs attachment seems to be mostly happening on the exopodic setae of the limbs, but also on the filter plates of the limbs 3 and 4 (Figs. 1A and 10). The trunk limbs attachment also seemed to touch trunk limb 5, but with a lower intensity than the “L5” attachment (compare Figs. 8B and 10B). Overall, attachment on the appendages remains difficult to clearly characterize due to their complex morphology.

Molting possibly affects all attachment sites

Observations of cuticle molts of the *D. magna* during the attachment test revealed that attached spores at the foregut and postabdominal attachment sites were shed by the animal along with the molt (Fig. 11A and B). As we did not observe a molt of rectum attachment, we do not know if spores attached to this part of the hindgut get shed. Observations of cuticle molts under a stereomicroscope revealed that the entire structure of the appendages, including the setae, also get shed by the *D. magna* (Fig. 11C and D).

References

Bento, G., P. D. Fields, D. Duneau, and D. Ebert. 2020. An alternative route of bacterial infection associated with a novel resistance locus in the *Daphnia–Pasteuria* host–parasite system. Heredity 1–11. Nature Publishing Group.

Duneau, D., P. Luijckx, F. Ben-Ami, C. Laforsch, and D. Ebert. 2011. Resolving the infection process reveals striking differences in the contribution of environment, genetics and phylogeny to host-parasite interactions. BMC biology 9:1–11.

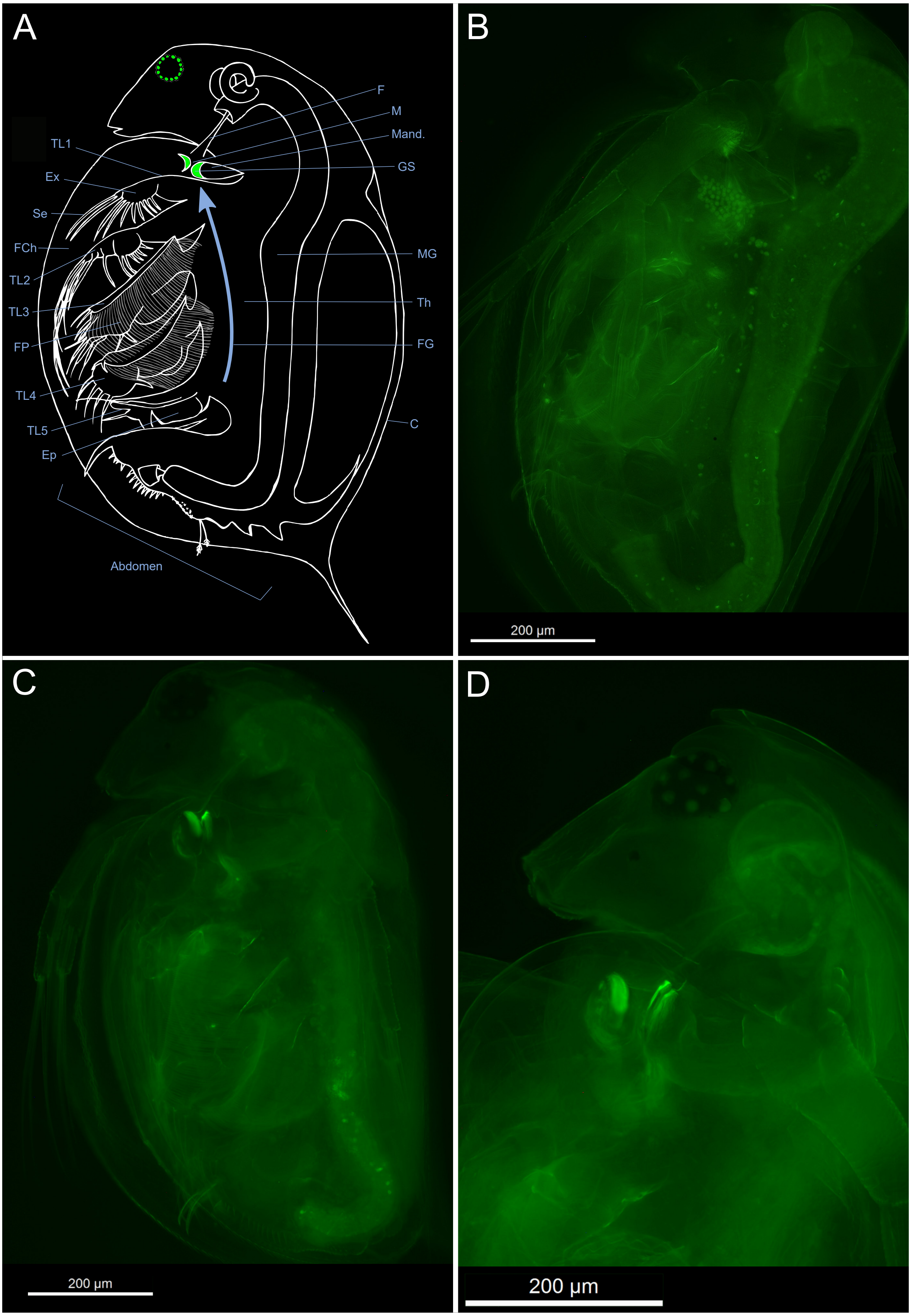

Figure 1 *Daphnia magna* female morphology. **A:** Structures relevant for the attachment test. Appendages morphology and structures names taken form (Fryer 1991). Going around the animal, from the upper left to the upper right: **TL1:** Trunk limb 1. Trunk limbs are also called thoracopods, thoracic appendages and phyllopods. **Ex:** Exopod of trunk limb 1. Exopods are present on all trunk limbs. **Se:** Setae of trunk limb 1. Setae are present on all trunk limbs, and are themselves covered with setules, also called spinules, not represented here. **FCh:** Filter chamber. **TL2 and 3** Trunk limbs 2 and 3. **FP:** Gnathobasic filter plate of trunk limb 3, also present on trunk limb 4. **TL4 and 5:** Trunk limbs 4 and 5. **Ep:** Epipodite of trunk limb 5, also present in the other trunk limbs. **Abdomen:** see Fig. 2. **C:** Carapace. **FG:** Food groove. The *Daphnia* filters the water, creating a water flow from the posterior part of the filter chamber to the anterior part where the mouth is. Particles are accumulated through the food groove to the mouth. **Th:** Thorax. **MG:** Midgut. **GS:** Grinding surface, autofluorescent in our samples. The grinding surface of the second mandible is represented, but not the rest of the mandible, as it is hidden behind. **Mand.:** Mandible. **M:** Mouth. **F:** Foregut, or oesophagous. **B:** Picture showing the trunk limbs of the *D. magna*. A few *P. ramosa* spores are visible in the gut, the food groove and on the expod of trunk limb 4. The fluorescent mass below the mandible is the supra-oesophagal ganglion, not spores. The antenna hides the foregut and the grinding surfaces of the mandibles. **C:** Picture showing the filter plates of *D. magna*. The foregut and mandible are also well visible. A few *P. ramosa* spores are visible in the distal part of the gut, one spore is visible on the filter plate. **D:** Picture showing the foregut and mandibles of *D. magna*.

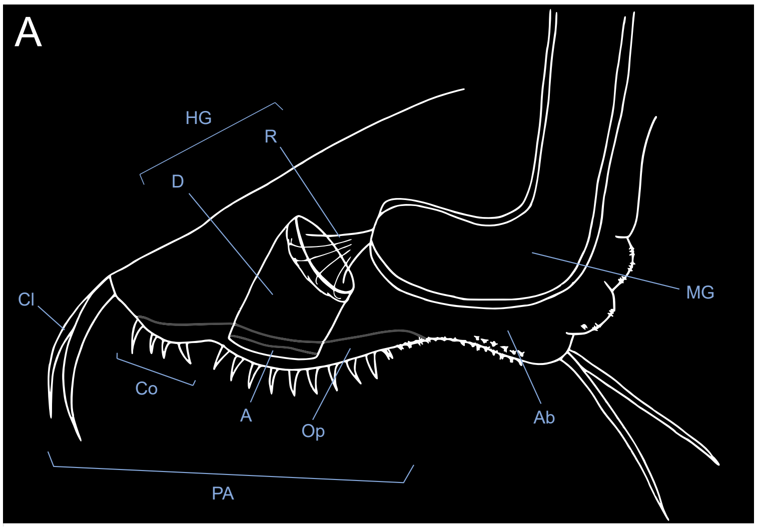

Figure 2 *Daphnia magna* female abdomen morphology. **A:** Structures relevant for the attachment test. Structures names taken form (Fryer 1991). A male abdomen is shown in Fig. 4C. Going around the abdomen, from the upper left to the upper right: **HG:** Hindgut. **R:** Rectum. The rectum is a flexible structure that links the midgut to the distal hindgut. It is represented here "folded" as the midgut and the distal hindgut are close to each other. However, the rectum can be elongated, as seen in Fig. 5C. **D:** Distal hindgut. **Cl:** Postabdominal claw. Setules present on the posterior part of the claws (not represented here) are visible in A to D. **Co:** Comb. The disposition of the two combs is specific to the species and the sex of the individual. The anus is positioned where the proximal larger comb is. **A:** Anus. **Op:** Postabdominal opening. **PA:** Postabdomen. **Ab:** Abdomen. **MG:** Midgut.

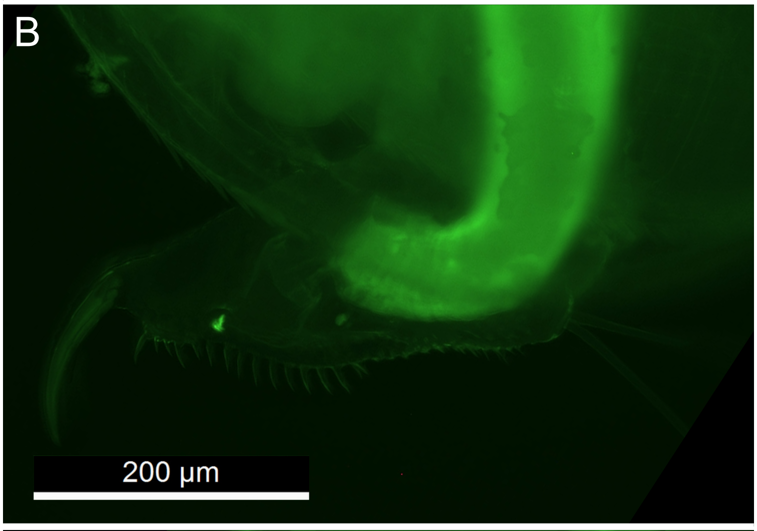

B: Picture showing the abdominal structures in *D. magna*. A few *P. ramosa* spores are visible on the anus.

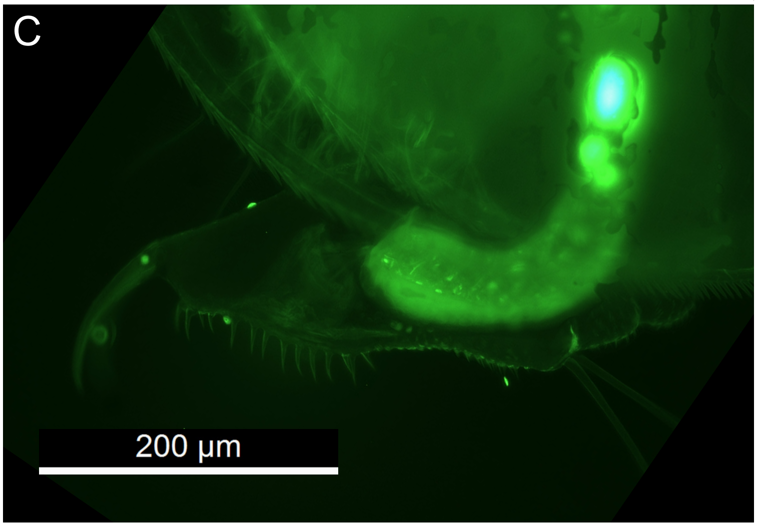

C: Picture showing the abdominal structures in *D. magna*. A few *P. ramosa* spores are visible on the external postabdomen.

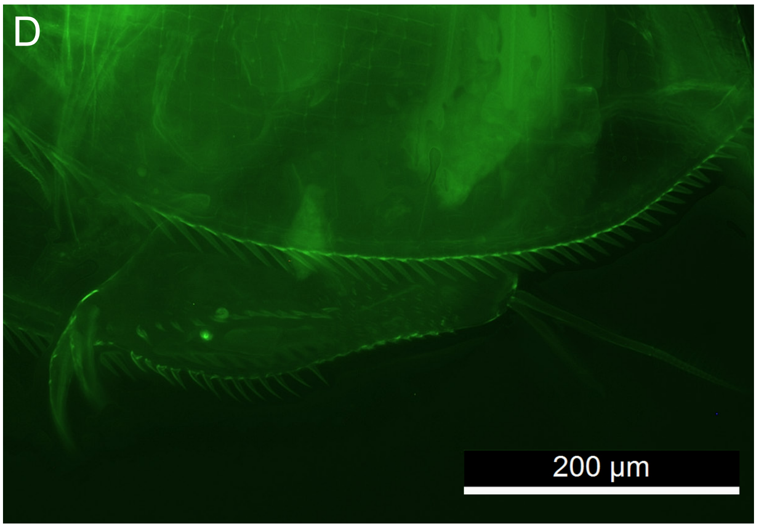

D: Picture showing the postabdominal opening and the anus from below in *D. magna*. One *P. ramosa* spore attaches to the larger postabdominal comb and one to the anterior part of the anus.

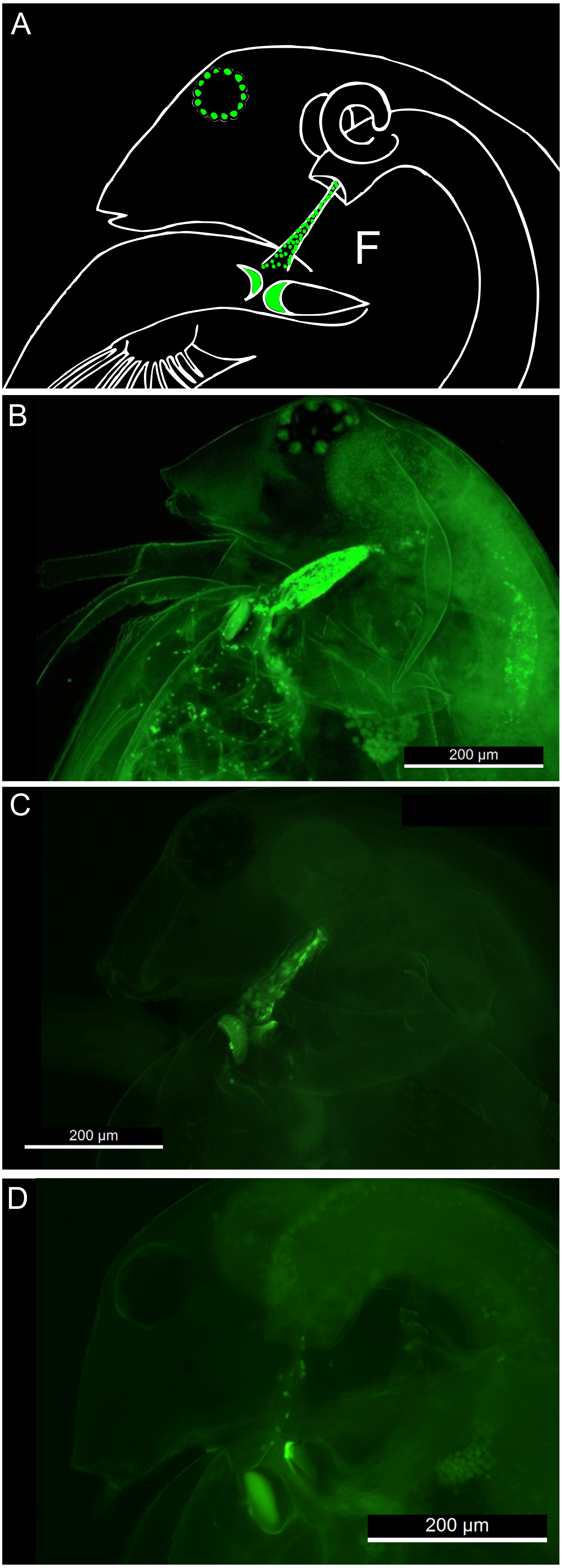

Figure 3 Foregut attachment "F" of *Pasteuria ramosa* in *Daphnia magna*.
A: Schematic view of the foregut attachment. The grinding surfaces on the mandibles autofluoresce. Spores of *P. ramosa* are represented as green dots. Spores cover the internal part of the foregut.

B: Picture showing the foregut attachment in *D. magna* with a high spore concentration. Spores also attach to the appendices in this sample.

C: Picture showing the foregut attachment in *D. magna* with a medium spore concentration.

D: Picture showing the foregut attachment in *D. magna* with a low spore concentration.

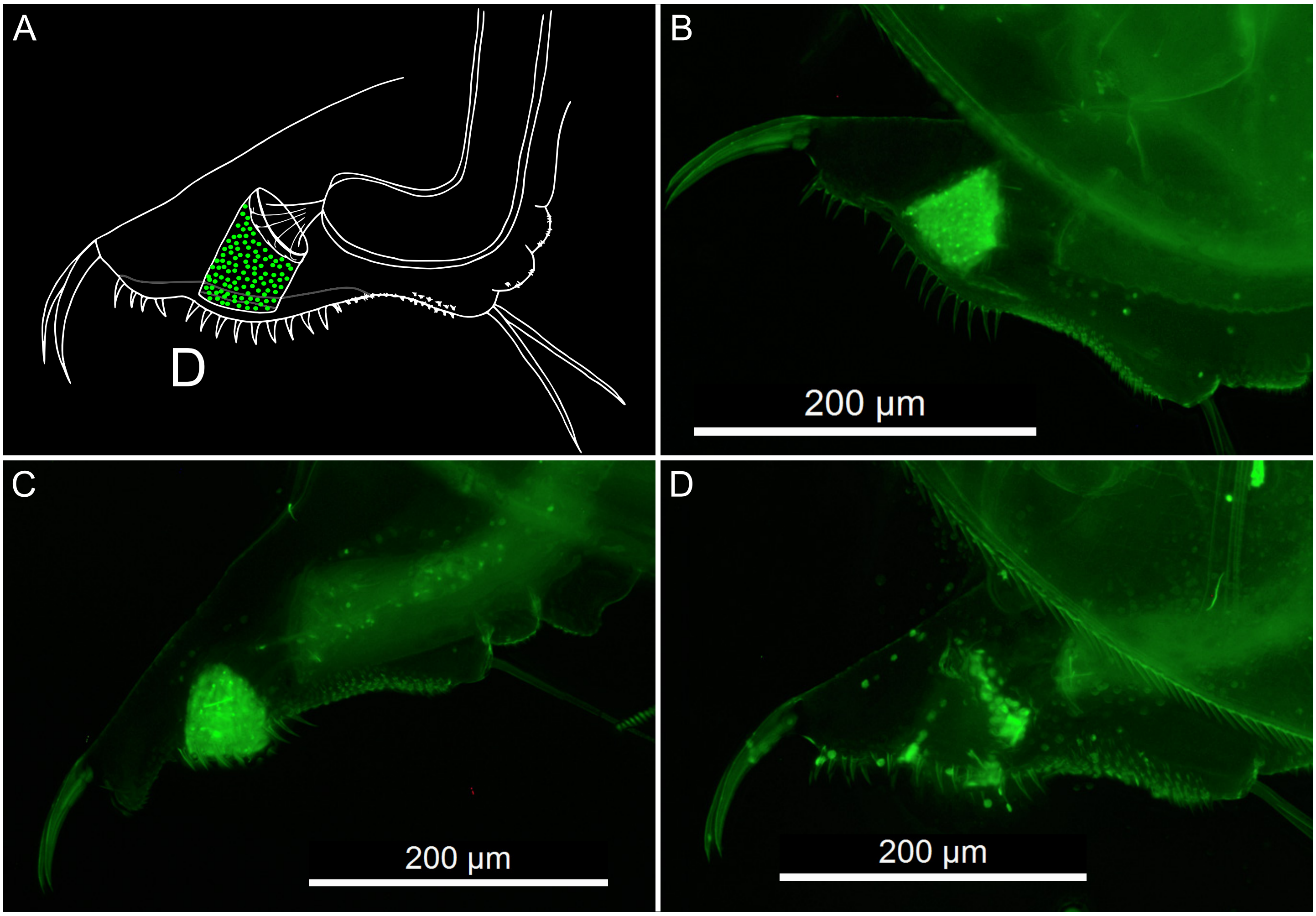

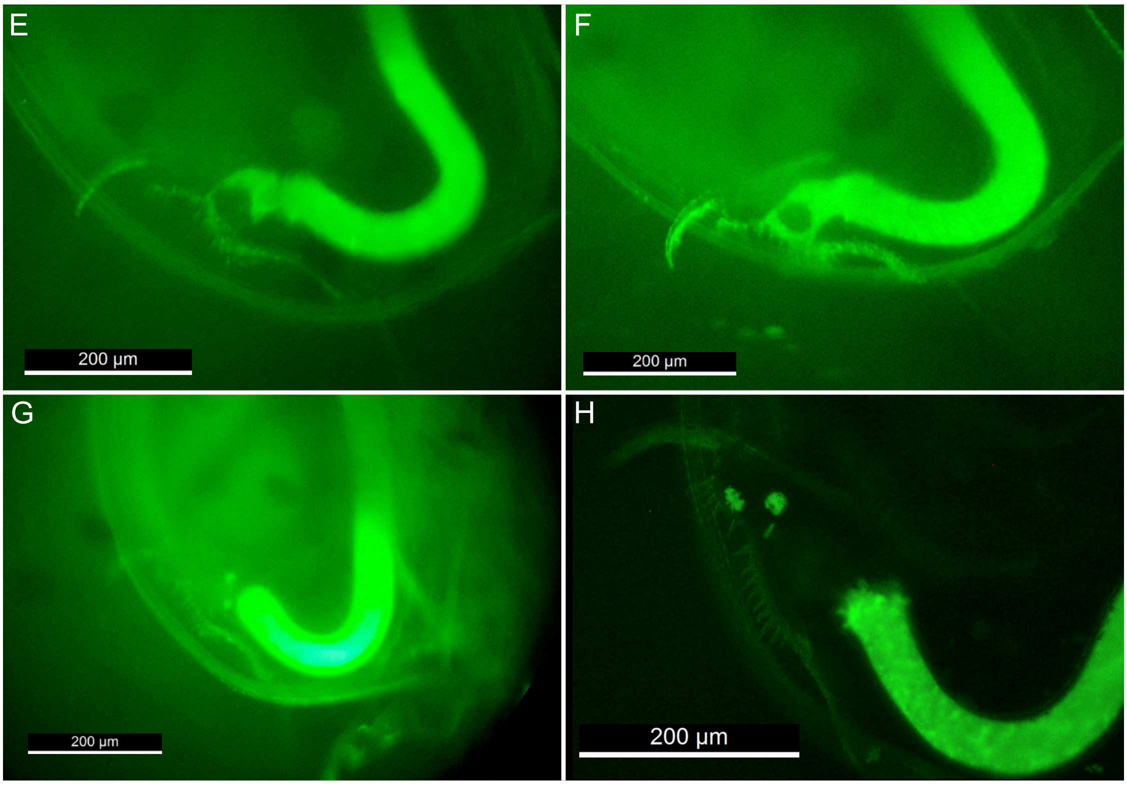

Figure 4 Distal hindgut attachment “D” of *Pasteuria ramosa* in *Daphnia magna*. **A:** Schematic view of the distal hindgut attachment. Spores of *P. ramosa* are represented as green dots. Spores cover the internal part of the distal hindgut. **B:** Picture showing the distal hindgut attachment in a female *D. magna*. **C:** Picture showing the distal hindgut attachment in a male *D. magna*. **D:** Picture showing a specific distal hindgut attachment in *D. magna*. Spores attach mostly at the corners of the distal hindgut. In this sample, spores also attach to the external postabdomen.

Remarkable patterns: E: Picture showing a specific distal hindgut attachment in *D. magna*. Spores attach to the rectum and to the proximal part of the distal hindgut. In this sample, spores also attach to the external postabdomen. Picture taken with a smartphone camera through a fluorescent-lens microscope LEICA DMI4000B. **F:** Picture showing a specific distal hindgut attachment in *D. magna*. Spores attach to the rectum and seem to attach to the medial part of the distal hindgut more than the lateral part. In this sample, spores also attach to the external postabdomen. Picture taken with a smartphone camera through a fluorescent-lens microscope LEICA DMI4000B. **G:** Picture showing a specific distal hindgut attachment in *D. magna*. Spores attach to proximal part of the distal hindgut, where it meets the rectum. Picture taken with a smartphone camera through a fluorescent-lens microscope LEICA DMI4000B. **H:** Possibly same as G, if the sample was slightly crushed and the distal hindgut was moved towards the distal part of the postabdomen. Alternatively, this could represent yet another attachment pattern not described in the present study. Another possibility would be that this pattern comes from an artefact.

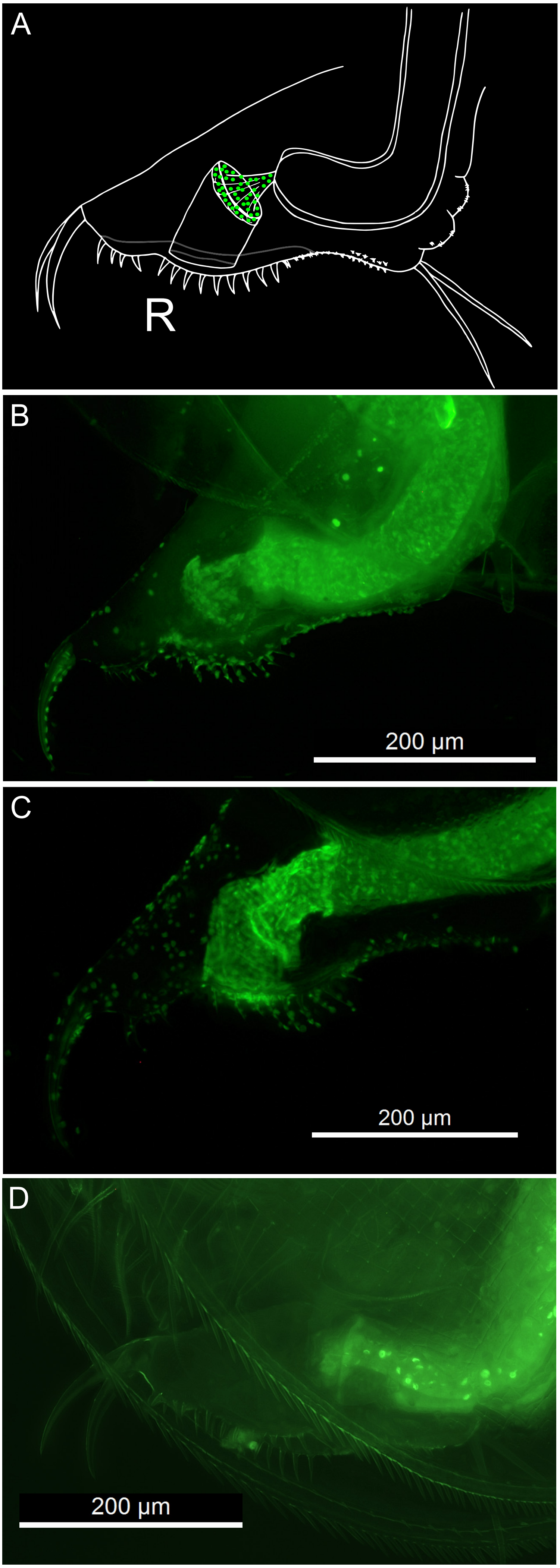

Figure 5 Rectum attachment "R" of *Pasteuria ramosa* in *Daphnia magna*.
A: Schematic view of the rectum attachment. Spores of *P. ramosa* are represented as green dots. Spores cover the internal part of the rectum.

B: Picture showing the "folded" rectum attachment. In this sample spores also attach to the external postabdomen.

C: Picture showing the "unfolded" rectum attachment. In this sample spores also attach to the external postabdomen.

D: Picture showing a possible artefact looking similar to the rectum attachment. In this sample it seems like the distal part of the midgut would be protruding from the midgut, making it look like a rectum attachment In this sample a few spores attach to the external postabdomen, on the larger comb.

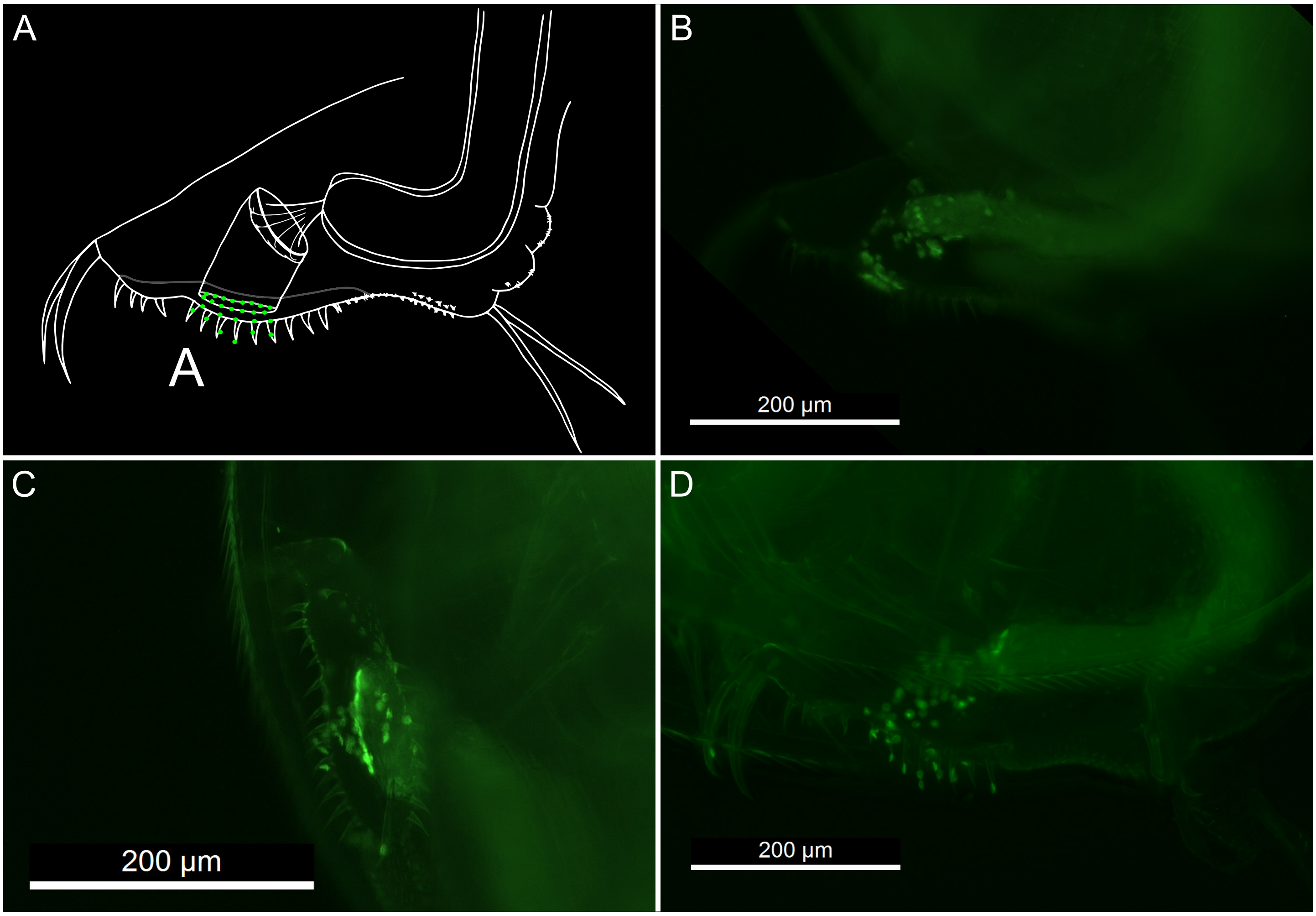

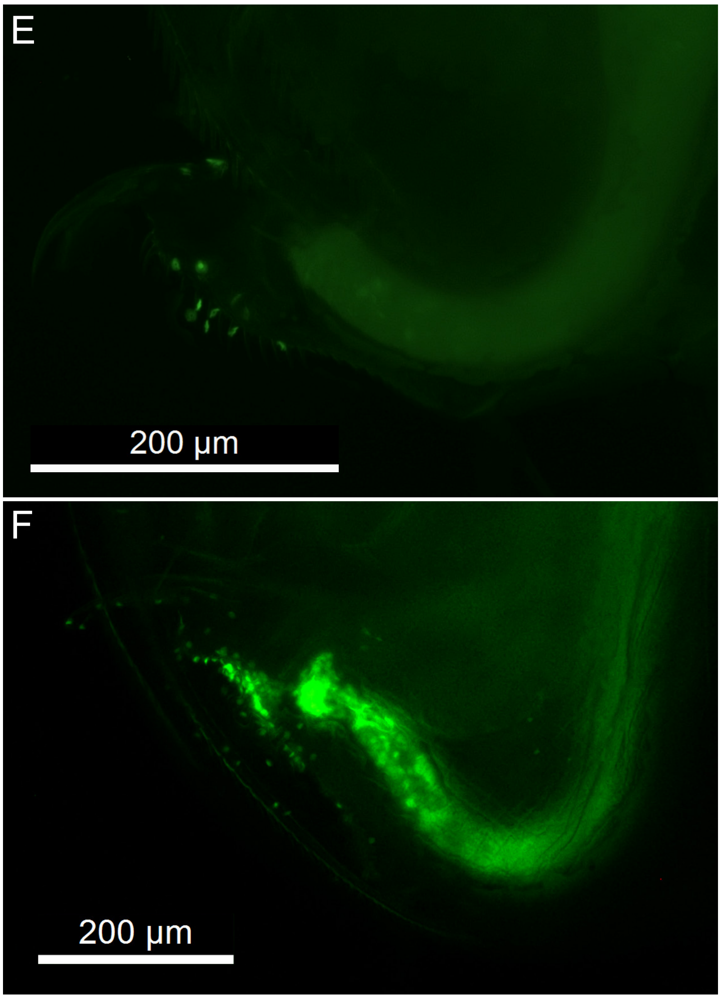

Figure 6 Anus attachment "A" of *Pasteuria ramosa* in *Daphnia magna*. **A:** Schematic view of the anus attachment. Spores of *P. ramosa* are represented as green dots. Spores attach to the anus and to the larger comb of the postabdomen. **B:** Picture showing the anus attachment. In this sample a few spores attach to the distal hindgut. **C:** Picture showing the anus attachment from below. **D:** Same as B, with a stronger attachment in the distal hindgut. One spore attaches to the postabdominal claw. **E:** Picture showing the anus attachment with a low spore concentration. In this sample a few spores attach to the external abdomen. **F:** Picture showing the anus attachment with a high spore concentration. In this sample spores also attach to the rectum and a few spores attach to the external abdomen. Picture taken with a reflex camera through a fluorescent-lens microscope LEICA DMI4000B.

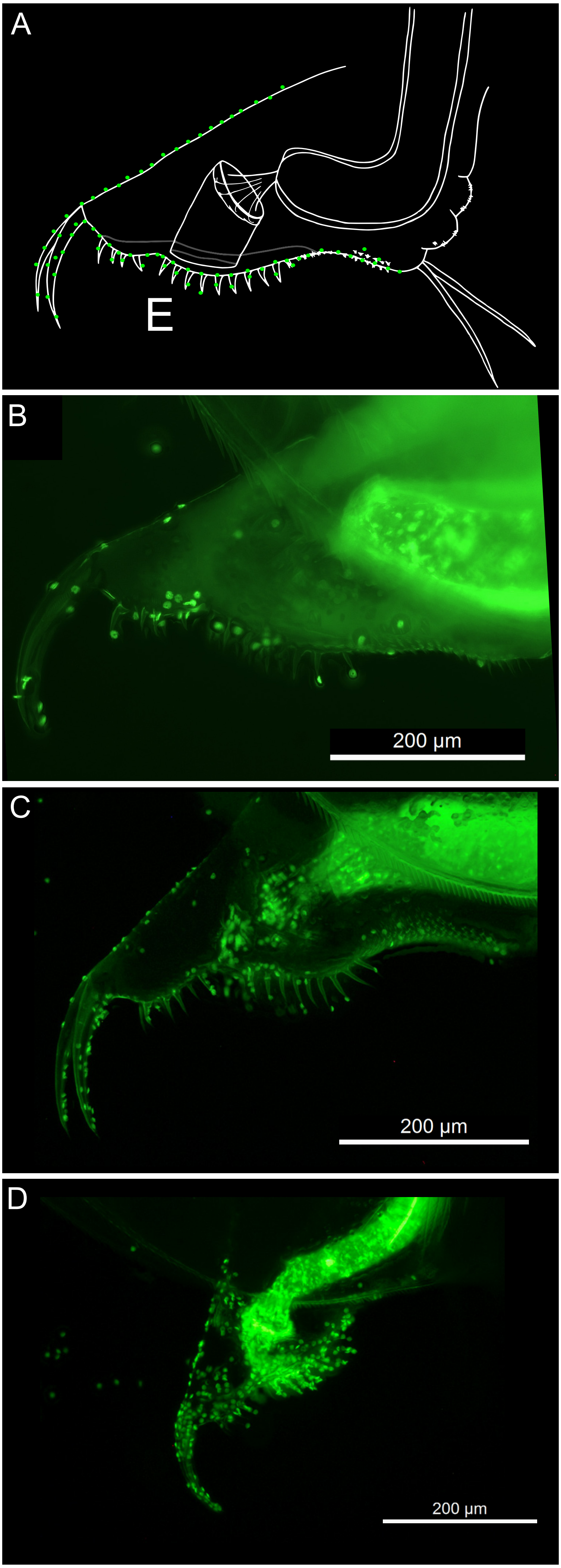

Figure 7 External postabdomen attachment "E" of *Pasteuria ramosa* in *Daphnia magna*. **A:** Schematic view of the external postabdomen attachment. Spores of *P. ramosa* are represented as green dots. Spores attach to the carapace outside of the postabdomen.

B: Picture showing the external postabdomen attachment with a low spore concentration.

C: Picture showing the external postabdomen attachment with a medium spore concentration. Note that spores attach more to the posterior part of the postabdominal claws, where the setules are (see Fig. 2). In this sample spores also attach to the rectum and the distal hindgut.

D: Picture showing the external postabdomen attachment with a high spore concentration. In this sample spores also attach to the rectum and the distal hindgut. Picture taken with a reflex camera through a fluorescent-lens microscope LEICA DMI4000B.

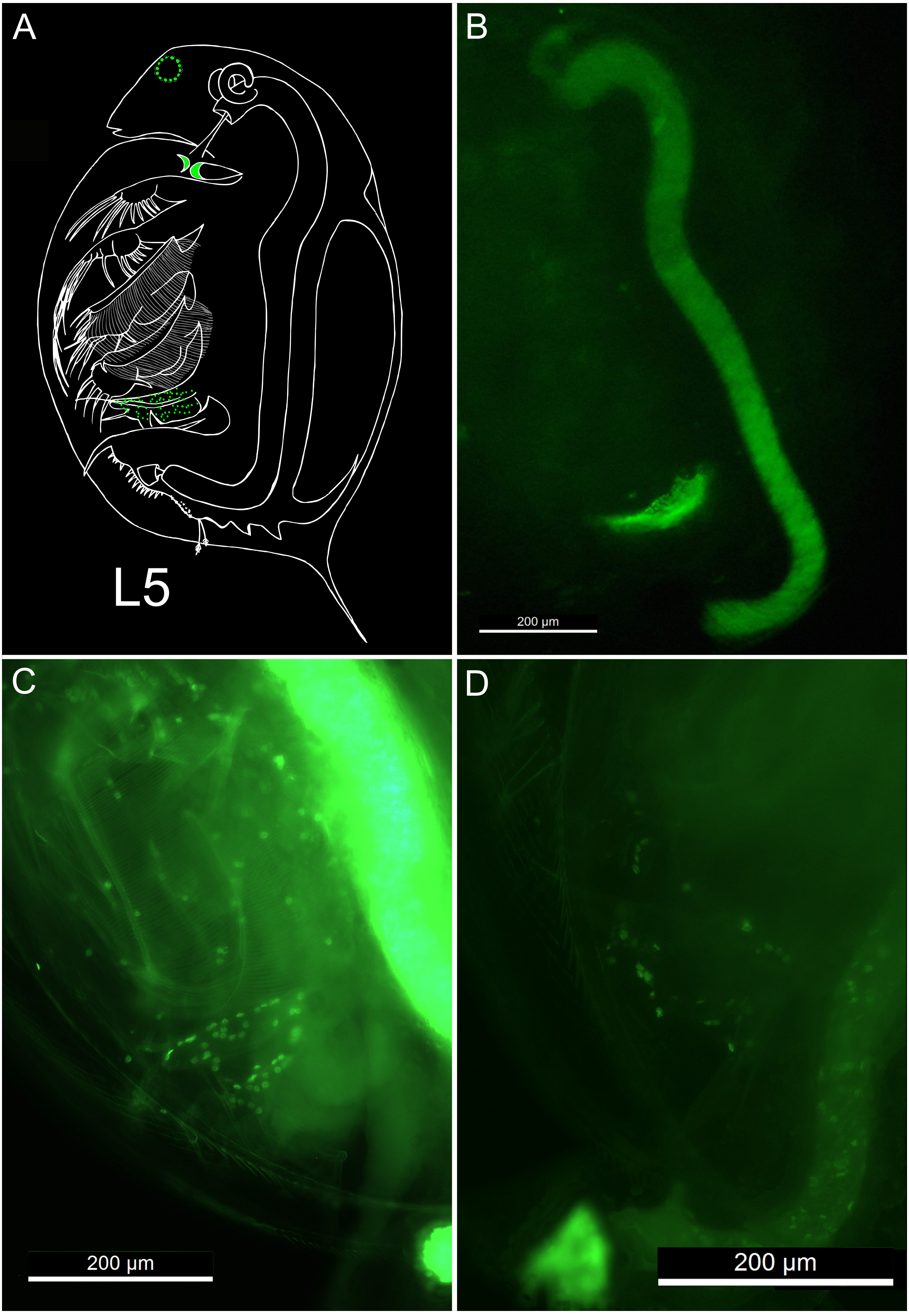

Figure 8 Trunk limb 5 attachment "L5" of *Pasteuria ramosa* in *Daphnia magna*. **A:** Schematic view of the trunk limb 5 attachment. Spores of *P. ramosa* are represented as green dots. **B:** Picture showing the trunk limb 5 attachment with a high spore concentration (10000 spores). Picture taken with a smart-phone camera through a fluorescent-lens microscope LEICA DMI4000B. **C:** Picture showing the trunk limb 5 attachment with a medium spore concentration (2000 spores). In this sample spores also attach to the filter plate. **D:** Picture showing the trunk limb 5 attachment with a medium spore concentration (2000 spores). In this sample spores also attach to the distal hindgut.

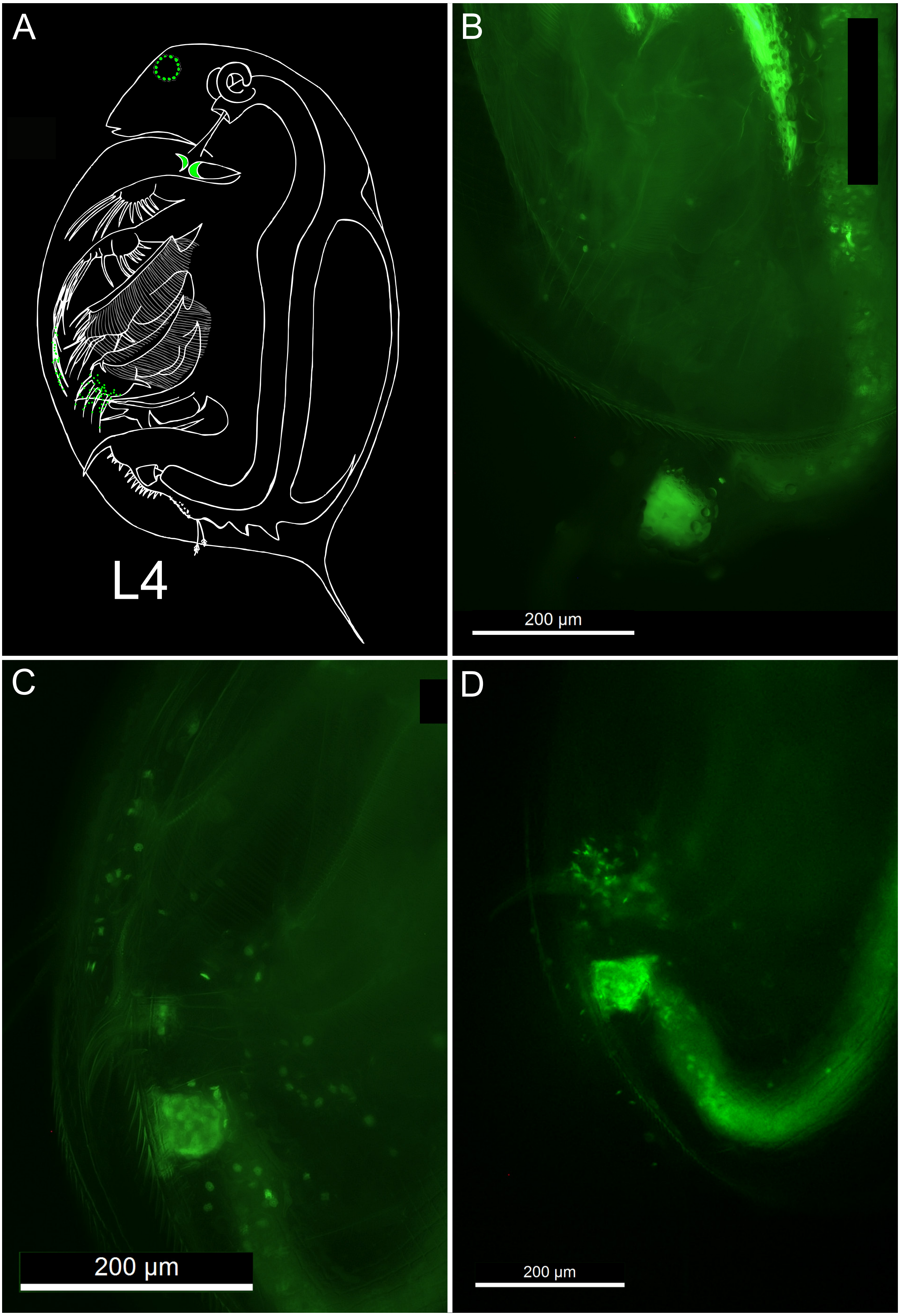

Figure 9 Trunk limb 4 attachment "L4" of *Pasteuria ramosa* in *Daphnia magna*. **A:** Schematic view of the trunk limb 4 attachment. Spores of *P. ramosa* are represented as green dots. **B:** Picture showing the trunk limb 4 attachment with a low spore concentration (500 spores). Spores are attached to the exopod of trunk limb 4 and to its setae. In this sample spores also attach to the distal hindgut. **C and D:** It is not clear where the spores attach, but they possibly attach to the distal part of the exopodic setae of the trunk limbs. In A, for clarity, we represent the trunk limbs separated from one other, but in reality, the trunk limbs overlap and exopodic setae from possibly all trunk limbs meet above the postabdominal claws. **C:** Picture showing the trunk limb 4 attachment with a medium spore concentration (2000 spores). In this sample spores also attach to the distal hindgut. **D:** Picture showing the trunk limb 4 attachment with a high spore concentration (10000 spores). In this sample spores also attach to the distal hindgut. Picture taken with a reflex camera through a fluorescent-lens microscope LEICA DMI4000B.

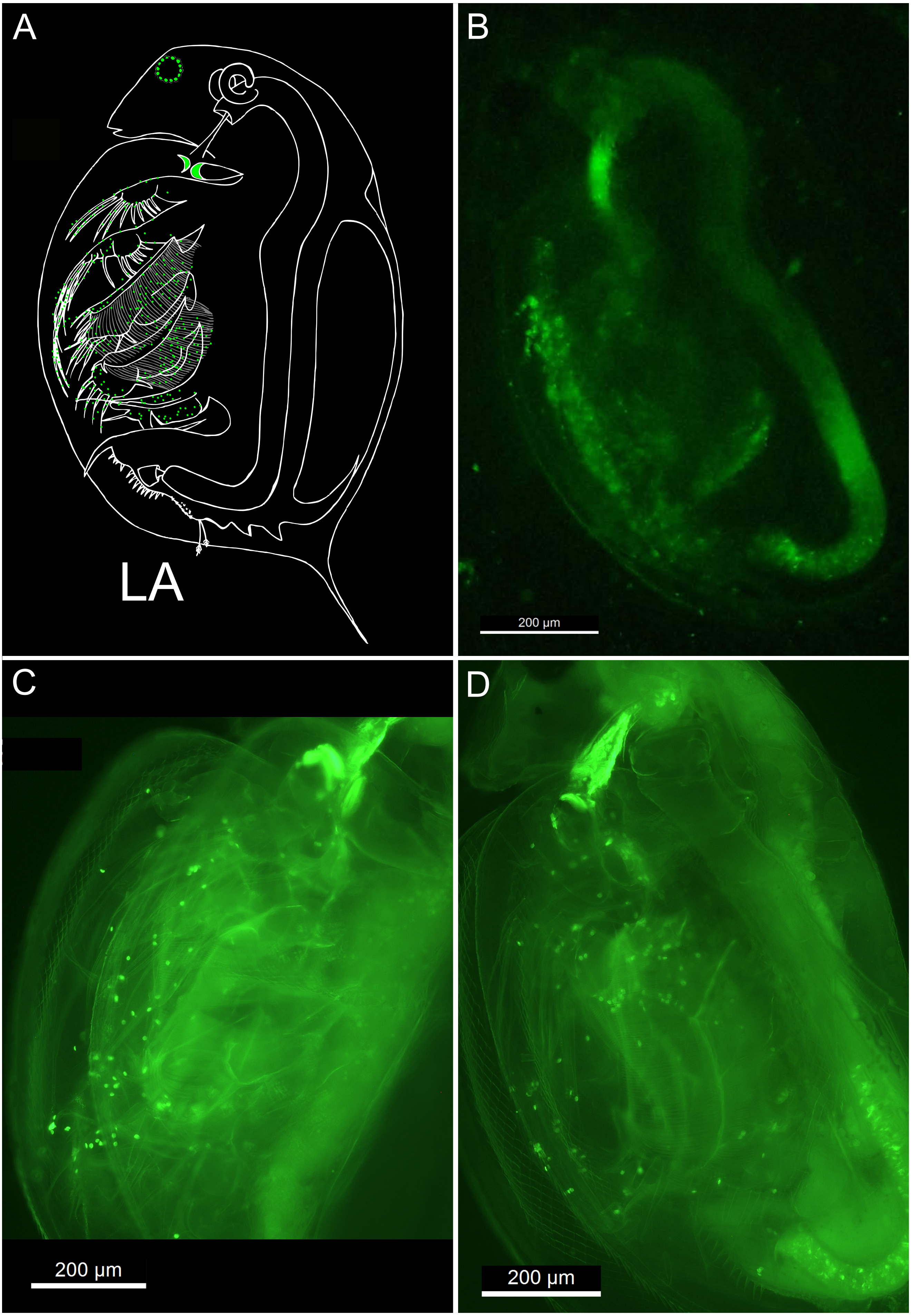

Figure 10 All trunk limbs attachment "LA" of *Pasteuria ramosa* in *Daphnia magna*. **A:** Schematic view of the trunk limbs attachment. Spores of *P. ramosa* are represented as green dots. Spores seem to attach to all trunk limbs, primarily on the exopodic setae and on trunk limb 5. **B:** Picture showing the trunk limbs attachment with a high spore concentration (10000 spores). Spores seem to attach to all trunk limbs, primarily on the exopodic setae and on trunk limb 5. In this sample spores also attach to the foregut. Picture taken with a reflex camera through a fluorescent-lens microscope LEICA DMI4000B. **C:** Picture showing the trunk limbs attachment with a medium spore concentration (2000 spores). Spores seem to attach to the exopodic setae of all trunk limbs. In this sample spores also attach to the foregut. **D:** Picture showing the trunk limbs attachment with a medium spore concentration (2000 spores). Spores seem to attach to the filter plates of trunk limbs 3 and 4. In this sample spores also attach to the foregut.

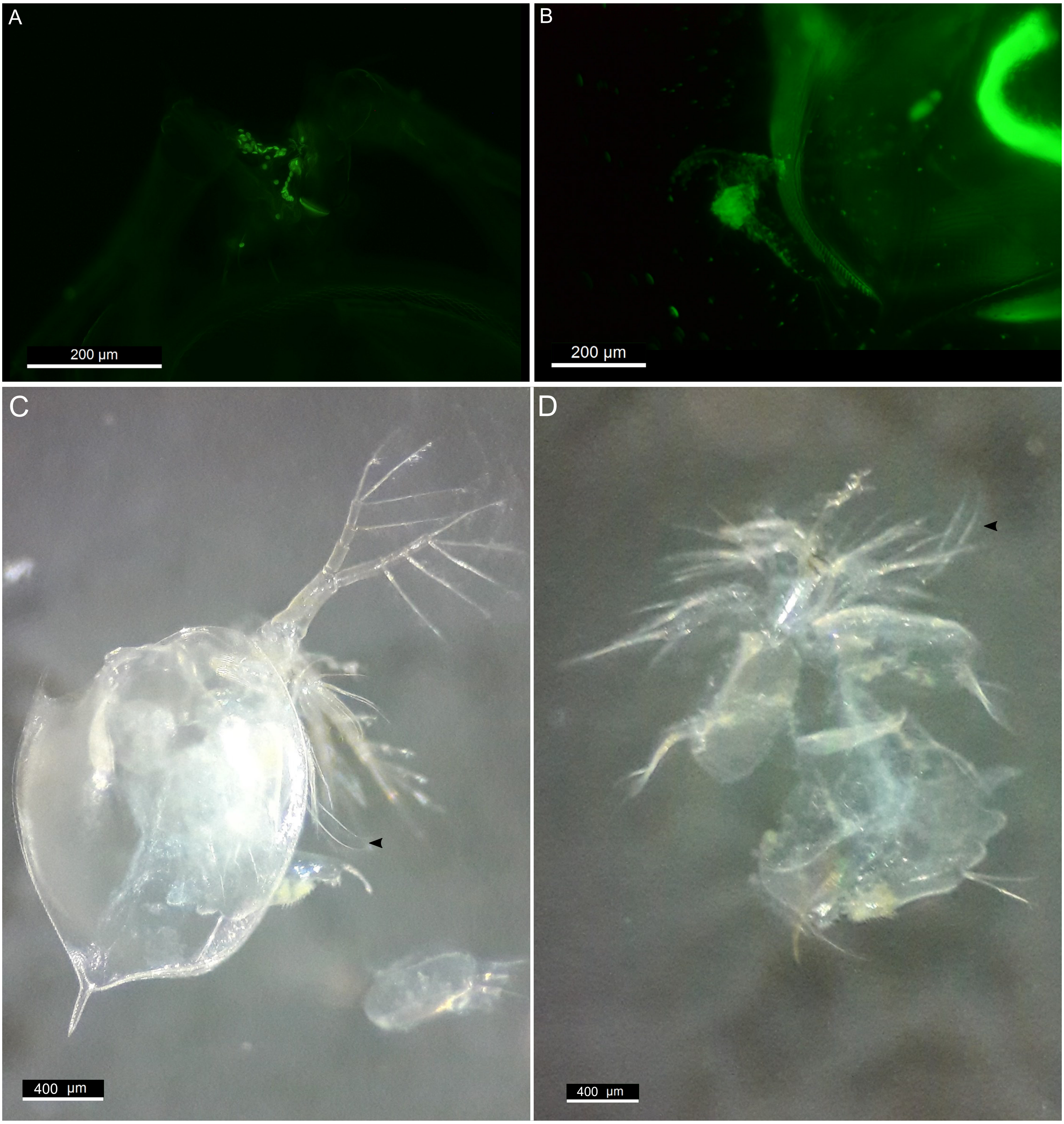

Figure 11 Cuticle molts in *Daphnia magna*. **A and B:** Cuticle molts in *Daphnia magna* with attached spores of *Pasteuria ramosa*. **A:** Picture of a cuticle molt of a foregut attachment with a low spore concentration. The foregut seems to have been split in two parts. The antennas are visible on both sides of the foregut, and one of the mandibles is visible at the bottom of the foregut. **B:** Picture showing the cuticle molt of a distal hindgut and external postabdomen attachment with a high spore concentration (10000 spores). The animal is in the process of molting and the gut is visible on the right side of the picture. Picture taken with a reflex camera through a fluorescent-lens microscope LEICA DMI4000B. **C and D:** Pictures showing the cuticle molt of entire *D. magna* individuals. Arrow heads point to the setae of the exopods. Pictures taken with a smartphone camera through a stereomicroscope.

Table 1 Details about the *Daphnia magna* genotypes used in the pictures of this supplementary. Attachment is described as a string of letters containing all sites where attachment was observed in one host individual. For example, in the CH-H-2015-9 host clone, we observed attachment on the external postabdomen (site E) in some repeats; and attachment on the postabdomen (site E), rectum (site R) and anus (site A) in other repeats. Previous scoring did not distinguish attachment on the distal hindgut (site D) from attachment on the rectum (site R). Hence, in some cases, we do not know if the observed attachment was "D" or "DR".

***D. magna* clone**		**Resistotype**										**Attachment site observed with high spore concentration**										**Comment**
**name**	**origin**	C1	C19	P15	P20	P21	P38	P39	P40	P41	P42	C1	C19	P15	P20	P21	P38	P39	P40	P41	P42	
CH-H-2015-9	Aegelsee	R	R	S	R	S	S	S	S	S	S	F	F	D or DR	F	D or DR	RE	E, REA	RE	RE	E, REA	NA
CH-H-2015-20	Aegelsee	R	R	S	R	S	S	S	S	S	S	F	F	D or DR	F	D or DR	RE	REA	REA	E, REA	RE	NA
CH-H-2015-35	Aegelsee	S	S	S	S	S	S	S	S	S	S	F	F	D or DR	F	D or DR	E	E, REA	E	E	E, RE	NA
CH-H-2015-36	Aegelsee	R	R	S	S	S	S	S	S	S	S	F	F	D or DR	F	D or DR	REA	REA	RE	REA, RE	REA, RE	NA
CH-H-2015-42	Aegelsee	R	R	S	S	S	S	S	S	S	S	F	F	D or DR	F	D or DR	RE	E, REA	REA	REA, RE	E, RE	NA
t1_10.3_2i_2	Aegelsee	R	R	R	R	R	S	S	S	S	S	F	F	D or DR	F	D or DR	REA, DE	E, REA, DE	REA, DE	DE	DE	P38 host
t1_10.3_2i_3	Aegelsee	R	R	R	R	R	S	S	S	S	S	F	F	D or DR	F	D or DR	EA	E, REA	E, REA, DE	E, RE	REA, DE	P39 host
t1_10.3_2i_6	Aegelsee	R	R	R	R	R	S	S	S	S	S	F	F	D or DR	F	D or DR	RE	DE, RE	REA, DE	E, DE, RE	E, REA	P40 host
t1_10.3_2i_11	Aegelsee	R	R	R	R	R	S	S	S	S	S	F	F	D or DR	F	D or DR	REA, EA, RE	E, REA	REA	REA, DE	DE, RE	P41 host
t1_10.3_2i_15	Aegelsee	R	R	R	R	R	S	S	S	S	S	F	F	D or DR	F	D or DR	REA	REA, DE	E, REA	REA	REA	P42 host
t2_17.3_4	Aegelsee	R	R	S	S	S	NA	NA	NA	NA	NA	NA	NA	D	FLA	DL4	NA	NA	NA	NA	NA	NA
t2_17.3_4i_12i_10	Aegelsee	R	R	S	S	S	NA	NA	NA	NA	NA	NA	NA	D	FLA	DL4	NA	NA	NA	NA	NA	NA
CH-H-2015-97	Aegelsee	S	S	S	S	S	NA	NA	NA	NA	NA	F	F	D	FLA	D	NA	NA	NA	NA	NA	NA
CH-H-2015-97i-6	Aegelsee	S	S	S	S	S	NA	NA	NA	NA	NA	F	F	D	FLA	D	NA	NA	NA	NA	NA	NA
RU-BOL-1	Russia	NA	NA	NA	NA	NA	NA	NA	NA	NA	NA	NA	NA	NA	NA	NA	NA	NA	NA	NA	NA	NA

Table 2 Details about the *Daphnia magna* – *Pasteuria ramosa* genotypes used in the pictures of this supplementary. Attachment is described as a string of letters containing all sites where attachment was observed in one host individual. Credits: CA: Camille Ameline, BH: Benjamin Hüssy, MF: Maridel Fredericksen.

Picture	*D. magna* clone	*P. ramosa* isolate	Resistotype	Attachment site	n spores	Dilution	Comment	Credits
Fig. 1B	CH-H-2015-36	P20	NA	NA	500	1:100	Picture used to show the anatomy. Trial picture to set the number of spores to use, too few spores were used to observe attachment.	CA
Fig. 1C	t1_10.3_2i_2	C1	R	F	500	1:100	**Host clone used to isolate P38**. Negative control, same as 2B.	CA
Fig. 1D	CH-H-2015-35	P38	S	E	500	1:100	Attachment not expected in the foregut, picture taken to show the anatomy of the foregut	CA
Fig. 2B	t1_10.3_2i_6	P39	S	A	1000	1;100	**Host clone used to isolate P40**. Too few spores to abserve sufficient attachment, picture used to show anatomy	CA
Fig. 2C			S	E	4000	1:100	Few spores visible on the external postabdomen, too few spores used, picture used to show anatomy	CA
Fig. 2D	t1_10.3_2i_2	P38	S	A	500	1;100	Too few spores to observe attachment, picture used to show anatomy of the postabdominal opening	CA
Fig. 3B	CH-H-2015-9	P20	S	F	500	1:10	The Daphnia was slightly crushed, which is why spores are observed below the mouth	BH
Fig. 3C	CH-H-2015-35	C1	S	F	2000	1;100	NA	CA
Fig. 3D	CH-H-2015-36	P20	S	F	1000	1:100	NA	CA
Fig. 4B	CH-H-2015-35	P21	S	D	500	1:10	NA	BH
Fig. 4C	CH-H-2015-35	P21	S	D	500	1:10	NA	BH
Fig. 4D	CH-H-2015-35	P41	S	DE	500	1:10	NA	BH
Fig. 4E	NA	NA	S	RDE	10000	1:10	NA	MF
Fig. 4F	NA	NA	S	RDE	10000	1:10	NA	MF
Fig. 4G	NA	NA	S	DE	10000	1:10	NA	MF
Fig. 4H	CH-H-2015-9	P20	NA	NA	10000	1:10	Rare pattern, not in the hindgut, migth be an artefact	BH
Fig. 5B	CH-H-2015-20	P41	S	RE	1000	1:10	NA	BH
Fig. 5C	CH-H-2015-35	P21	S	DRE	1000	1:10	NA	BH
Fig. 5D	t1_10.3_2i_2	C1	NA	NA	1000	1;100	Possible artefact that looks like R attachment	CA
Fig. 6B	t1_10.3_2i_6	P39	S	AD	4000	1;100	NA	CA
Fig. 6C	t1_10.3_2i_6	P40	S	A	4000	1;100	NA	CA
Fig. 6D	t1_10.3_2i_2	C1	S	DA	4000	1:100	NA	CA
Fig. 6E	CH-H-2015-42	P39	S	AE	4000	1;100	NA	CA
Fig. 6F	t1_10.3_2i_2	P41	S	RAE	10000	1:10	Picture taken of a moving *Daphnia* with a camera through the ocular in a standard attachment test	CA
Fig. 7B	t1_10.3_2i_6	P42	S	E	4000	1;100	NA	CA
Fig. 7C	CH-H-2015-9	C19	S	RDAE	4000	1;10	NA	BH
Fig. 7D	NA	NA	S	RDAE	4000	1:10	NA	BH
Fig. 8B	RU-BOL-1	4048	S	L5	10000	1;10	Picture taken of a moving *Daphnia* with a camera through the ocular in a standard attachment test	MF
Fig. 8C	RU-BOL-1	4048	S	L5	4000	1;100	NA	CA
Fig. 8D	CH-H-2015-97i-6	P21	S	DL5	10000	1:100	NA	CA
Fig. 9B	t2_17.3_4	P21	S	DL4	10000	1:100	NA	CA
Fig. 9C	CH-H-2015-97i-6	P21	S	DL4	10000	1:100	NA	CA
Fig. 9D	CH-H-2015-36	P15	S	DRL4	10000	1:10	NA	CA
Fig. 10B	t2_17.3_4i_12i_10	P20	S	FLA	10000	1:10	Picture taken of a moving *Daphnia* with a camera through the ocular in a standard attachment test	CA
Fig. 10C	CH-H-2015-97i-6	P20	S	FLA	10000	1:100	NA	CA
Fig. 10D	t2_17.3_4	P20	S	FLA	10000	1:100	NA	CA
Fig. 11A	CH-H-2015_97	P20	S	F	1000	1:100	Moult	CA
Fig. 11B	NA	NA	S	DE	10000	1:10	Picture taken of a moving *Daphnia* with a camera through the ocular in a standard attachment test	CA
Fig. 11C	NA	NA	NA	NA	NA	NA	Moult, picture taken through the ocular of a stereomicroscope with normal light	MF
Fig. 11D	NA	NA	NA	NA	NA	NA	Moult, picture taken through the ocular of a stereomicroscope with normal light	MF
